# Island influences on plant functional traits and trait–trait associations across species‐ and community‐scales

**DOI:** 10.1111/nph.71040

**Published:** 2026-02-27

**Authors:** Yanjun Song, Nate G. McDowell, Zengke Zhang, Alexandria L. Pivovaroff, Mingshan Xu, Wentao Ren, Dong He, En‐Rong Yan, Sylvain Delzon

**Affiliations:** ^1^ School of Biological Sciences Washington State University Pullman WA 99164‐4236 USA; ^2^ Zhejiang Zhoushan Island Observation and Research Station, Tiantong National Forest Ecosystem Observation and Research Station, and Shanghai Key Lab for Urban Ecological Processes and Eco‐Restoration, School of Ecological and Environmental Sciences East China Normal University Shanghai 200241 China; ^3^ UMR BIOGECO INRAE, Univ. Bordeaux Pessac 33615 France; ^4^ Atmospheric Sciences and Global Change Division Pacific Northwest National Lab Richland WA 99354 USA; ^5^ Institute of Eco‐Chongming (IEC) Shanghai 200241 China; ^6^ Biology Department Occidental College Los Angeles CA 90041 USA; ^7^ Department of Geography and Spatial Information Techniques Ningbo University Ningbo 315211 China; ^8^ College of Ecology and the Environment Xinjiang University Urumchi 830046 China

**Keywords:** Archipelagos, community, hydraulics, island syndrome, shared island–mainland species, the island rule, trait association, wood density

## Abstract

- The island rule predicts gigantism or dwarfism in body size of island species relative to their mainland counterparts. However, whether other functional traits shift and whether trait–trait associations on islands differ between species and community levels remains unclear.
- We measured 13 carbon‐ and water‐related functional traits in 37 shared tree species across 35 eastern Chinese islands and 66 nearby mainland plots. We examined species‐level trait value shifts and associations under the island rule and compared trait associations between species and communities.
- Most size‐related, wood‐anatomical, and hydraulic traits shifted on islands, with large values decreasing and small values increasing; yet, their associations remained stable, aligning with the global trait spectrum and trait–trait coevolution. This stability, despite trait value shifts, suggests evolutionary integration of functional strategies. By contrast, island community‐scale trait associations diverged from shared species‐level patterns and sometimes reversed, such as positive relationships between wood density and resource‐acquisitive traits. Community‐level trait associations were stronger on islands, likely reflecting constrained environmental filtering and migration limitation.
- These contrasting patterns suggest that dominant species can restructure trait associations at the community level, with implications for ecosystem functioning and carbon storage, thereby advancing understanding of plant trait strategies in island systems.

## Introduction

Islands are natural laboratories for examining how functional traits and species strategies are shaped by their evolutionary history and environmental gradients (Whittaker *et al*., 2017; Taylor *et al*., 2019). Plant functional traits affect growth, survival, and fitness by influencing resource uptake (e.g. carbon, water, and nutrients) and stress tolerance, thereby offering key insights into ecological strategies (Wright *et al*., 2004; Violle *et al*., 2007; Poorter *et al*., 2018). Here, we use a trait‐based framework to investigate growth strategies of island plants.

Trait‐based approaches have advanced island biogeography, particularly through the lens of the ‘island rule’ – a concept originally developed for animals but increasingly applied to plants (Burns, 2019). The island rule predicts that small species exhibit insular gigantism and large species exhibit insular dwarfism relative to mainland counterparts, converging toward intermediate sizes across species (Foster, 1964). These size shifts result from different selective pressures, including predation, altered competition, resource availability, and unique abiotic conditions such as typhoons and strong solar radiation, and evolutionary drift (Yamashita *et al*., 2000; Lomolino, 2005; Biddick & Burns, 2021). Geographic isolation typically facilitates divergence, with environmental filtering and selection pressures in insular systems shaping trait evolution (Carlquist, 1974; Ottaviani *et al*., 2020). Dwarfism typically evolves in resource‐limited or stressful environments (e.g. nutrient‐poor soils, strong winds, deep shade), whereas gigantism becomes more common in which herbivore pressure is reduced and resources are relatively abundant (Burns, 2019; Biddick & Burns, 2021). Consequently, island species evolve body sizes that optimize survival and resource‐use efficiency under local ecological constraints.

Although size‐related morphological shifts in plant height, flower size, and leaf area are well‐documented (Biddick *et al*., 2019; Hendriks, 2019; Biddick & Burns, 2021), recent studies suggest that physiological traits also respond to insular conditions (Ottaviani *et al*., 2020; Schrader *et al*., 2021). The concept of gigantism and dwarfism does not translate linearly from size‐related traits to physiological traits. However, the concept that traits may shift toward more conservative values associated with greater stress, or more acquisitive values under the release of competitive or herbivorous pressure, is logical. Traits such as wood density (WD), leaf dry matter content (LDMC), and xylem conduit diameter reflect fundamental ecological strategies in drought tolerance, carbon conservation, and hydraulic efficiency. On islands, reduced herbivory, limited resources, and buffered microclimates can affect these strategies (Carlquist, 1974; Schrader *et al*., 2021; Zizka *et al*., 2022). For instance, increased WD and LDMC enhance structural support and water conservation, reflecting conservative resource‐use strategies favored under limited resource availability (Reich, 2014). The evolution of insular woodiness has been linked to drought adaptation and reduced disturbance (Zizka *et al*., 2022), indicating that insular effects extend beyond body size to other functional traits. Considering functional trait strategies thus offers a more mechanistic understanding of insular divergence and trait evolution under geographic isolation.

Despite growing interest in insular trait shifts, trait associations remain underexplored in island systems (Ottaviani *et al*., 2020). Trait associations are critical for understanding plant ecological strategies and trade‐offs, particularly along the conservative‐acquisitive spectrum of resource use (Reich, 2014; L. D. Anderegg *et al*., 2018). Global trait spectra describe conserved interspecific trait–trait relationships across vegetation types and spatial scales, providing a reference framework for evaluating trait associations (Wright *et al*., 2004; Chave *et al*., 2009; Reich, 2014). Traits related to carbon and water acquisition, such as leaf, stem, and root traits, often covary in predictable ways aligned with plant growth rates and mortality risk, with acquisitive species typically exhibiting high photosynthetic capacity and hydraulic conductance (Kong *et al*., 2019; Song *et al*., 2022). This conservative‐acquisitive spectrum links coordinated trait variation to functional strategies underlying plant growth, survival, reproduction, and fitness (Wright *et al*., 2004; Chave *et al*., 2009; Reich, 2014; Díaz *et al*., 2016). Studying trait associations in insular contexts can therefore reveal how plants balance functional trade‐offs under migration limitations and environmental filtering.

If insular trait shifts follow the island rule, associated trait relationships may also shift, either due to the decoupling of previously linked traits or as an adaptation to island‐specific selective pressures. Alternatively, stable trait associations despite trait shifts may indicate strong functional coregulation governing trade‐offs between resource conservation and acquisition. Although recent studies have shown that certain species‐level trait shifts follow the island rule (Biddick *et al*., 2019; Benítez‐López *et al*., 2021; Biddick & Burns, 2021) and a few have explored trait associations (Burns, 2016; Ciarle, 2024; Midolo *et al*., 2024), it remains understudied whether these size‐shifts in traits also influence species‐level trait associations. Importantly, trait associations also differ between organizational scales. Species‐level trait correlations can weaken, strengthen, or even reverse at the community level, shaped by community assembly processes and species dominance (Ackerly *et al*., 2002; Vile *et al*., 2006; Kichenin *et al*., 2013; L. D. Anderegg *et al*., 2018). Community‐weighted mean (CWM) traits, influenced by the functional traits and relative abundances of coexisting species (Ackerly & Cornwell, 2007), provide strong predictors of ecosystem functioning and offer scalable insights for modeling productivity and biogeochemical fluxes (Vile *et al*., 2006; Trugman *et al*., 2020; Anderegg *et al*., 2024). Given that island systems often support lower species richness and reduced functional diversity (Whittaker & Fernández‐Palacios, 2007; Russell & Kueffer, 2019; Whittaker *et al*., 2023), they typically occupy a narrower range of trait variation (Schrader *et al*., 2023). Consequently, species‐ and community‐level trait associations may be more aligned on islands than on the mainland. To our knowledge, no studies have investigated trait associations at the species or community levels in insular systems.

**Fig. 1:**
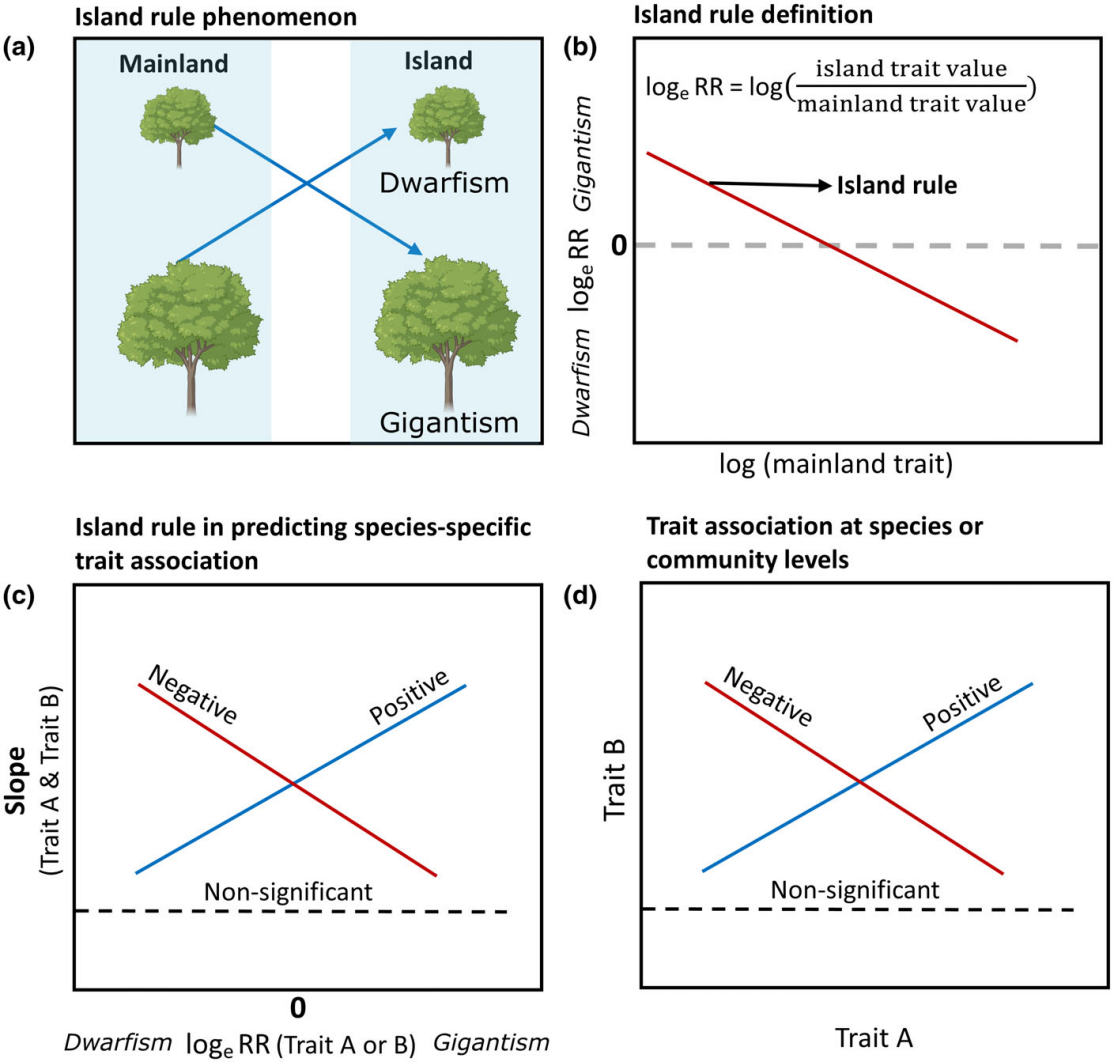
Conceptual figure showing the island rule and trait associations. (a) The island rule: species with small trait values increase (gigantism), and species with large trait values decrease on islands (dwarfism). (b) Definition by regressing log_e_ RR = log(insular trait value/mainland trait value) against natural‐log‐transformed mainland trait values; slope < 0 indicates trait shifts follow the island rule, while slope = 0 indicates no conformity. (c) The island rule predicting species‐level pairwise trait association in island plants. (d) Trait associations comparison at species and community‐weighted mean (CWM) levels in island plants. Tree pictures were obtained from ‘BioRender’ (https://BioRender.com/xtcjemq).

Here, we investigate how trait shifts and trait associations differ between island and mainland woody plants at both species and community levels. We measured 13 functional traits related to carbon and water use in > 3000 individuals representing 153 species across 35 eastern islands in China. These were compared with 66 mainland plots covering 37 shared species. Specifically, we address three overarching questions: (1) Do plant functional traits follow the island rule shifts, becoming smaller on islands if the mainland *values* are high and vice versa (Fig. 1a)? (2) Across species, do insular shifts in trait A (i.e. log_e_ RR; Fig. 1b) affect species‐specific within‐island A‐B regression slope (β, estimated from individual‐level data; Fig. 1c)? (3) Do trait associations quantified across shared species translate to community‐level trait associations (Fig. 1d)? We have three hypotheses. First, in more stressful insular environments, species with acquisitive carbon‐ and water‐related trait *values* will shift toward more conservative *values*, whereas species with more conservative values may shift toward acquisitive values as insular conditions could relax constraints (e.g. reduced disturbance or enemy pressure). Second, across species, insular trait shift (log_e_ RR in trait A) affects its associated species‐specific within‐island slope β (A‐B), indicating that insular trait divergence covaries with shifts within‐species trait associations. Third, community‐level trait associations will be stronger than shared‐species trait associations, because differences in species composition across communities reinforce coordinated shifts in community‐average traits.

## Materials and Methods

### Study sites

We conducted this study during the summer of 2016 and 2017 on 35 eastern Chinese islands (25.45°–37.98° N, 119.28°–122.78° E; Supporting Information Fig. S1). Annual temperatures range from 11.4°C (Beichangshan) to 19.9°C (Dayu), and annual precipitation from 487 mm (Beichangshan) to 1411 mm (Fenghuo). Forests are mainly composed of deciduous and evergreen broad‐leaved species, with a few needle‐leaf conifers (pines). Islands are characterized by more nutrient‐poor soils and more arid climates than the mainland (Zhang *et al*., 2025). The majority of islands (28 of 35) are located within the Zhoushan Archipelago (Fig. S1), which remained connected to the mainland until the mid‐Holocene (*c*. 8500 yr; Zhang *et al*., 2025), implying a shared ancestral species pool at the time of isolation (Xu *et al*., 2017). Although subsequent colonization, local extinction, and *in situ* differentiation may have modified community composition, dominant vegetation types remain broadly similar between mainland and Zhoushan Archipelago.

We established 150 plots (20 × 20 m quadrates for forests or 10 × 10 m quadrates for shrubs) across these islands (Table S1; Fig. S1), spanning 12° latitude over a transect *c*. 300 km wide (west–east) and *c*. 1600 km long (south–north), across a wide humidity gradient (aridity index 0.38–1.16). We recorded geographic coordinates for each island. Island sizes range from 0.006 to 409.9 km^2^, with distances to the nearest mainland between 0.65 and 77.0 km. Within each plot, we tagged all woody individuals with height ≥ 1 m, measured diameter at breast height (i.e. 1.3 m), tree height (H, m), and identified species. We used the 1‐m‐height threshold because individuals above this size account for over 90% of woody species richness on eastern islands, effectively capturing most local woody plant biodiversity (Xu *et al*., 2023). For comparison, we used data from 66 nearby mainland plots (Fig. S1) containing 37 species shared with the islands (Table S2). For both island and mainland sites, we extracted climate data related to water availability, temperature, wind, and light from WordClim v.2.0 (Methods S1) using site coordinates. We measured soil carbon (C), nitrogen (N), and phosphorus (P) at each site (Methods S2). Thirty‐seven of the total 153 island species were identified based on the presence of both island‐ and mainland‐endemic species, and on limitations in field sampling. Although mainland plots could not capture all island species, this approach was both sufficient and representative, as the selected plots formed the largest and most complete subset covering all shared species.

### Trait measurements

Due to the natural distribution and limited abundance of some species within plots, we sampled ≥ three healthy individuals per species per plot for functional trait measurements. This approach follows the minimum recommended standard for species‐level trait characterization in field studies under logistical constraints (Perez‐Harguindeguy *et al*., 2013). Many species occurred in multiple plots, resulting in a total sample size of ≥ 5 individuals per species across the study. This sampling effort is sufficient to characterize species‐level mean trait values, while we acknowledge that it provides limited resolution of intraspecific variability. Specifically, we harvested the three branches (*c*. 50 cm) on the most illuminated side with an average height of 4 m to reduce the phenotypic variation among individuals per plot. For each branch per individual, we collected 13 traits (Table 1; Fig. S2) related to plant size and toughness (four traits), wood anatomy and hydraulics (six traits), and branch size and density (three traits). The four traits related to plant size and toughness included plant height (H, m), individual mean leaf area (MLA, cm^2^), specific leaf area (SLA, cm^2^), and leaf dry matter content (LDMC, g g^−1^). Tree height, SLA, and MLA indicate light capture capacity, and LDMC refers to structural toughness (Poorter *et al*., 2018). We used a Vertex meter (Vertex‐IV, Haglöf Haglof, Dalarna, Sweden) to measure tree height. To quantify MLA, SLA, and LDMC, we randomly selected 20 healthy and mature leaves and measured leaf area using a leaf area meter (Li3‐100; Li‐Cor Biosciences, Lincoln, NE, USA). Afterward, all leaves were weighed and then dried at 75°C for 48 h to determine dry weights. MLA was calculated as total leaf area divided by leaf number, and SLA was calculated as leaf area divided by leaf dry mass. LDMC was determined as leaf dry weight over leaf fresh weight.

**Table 1:** Overview of 13 functional traits collected from eastern island sites and mainland sites in this study.

Traits classification	Trait name (Abbreviation)	Unit	No. of island sites	No. of mainland sites	No. of shared species	Tested hypotheses (H)
Size and toughness	Tree height (H)	m	35	66	50	H1, H2, H3
	Mean leaf area (MLA)	cm^2^	35	66	37	H1, H2, H3
	Specific leaf area (SLA)	cm^2^ g^−1^	35	66	37	H1, H2, H3
	Leaf dry matter content (LDMC)	g g^−1^	35	66	37	H1, H2, H3
Hydraulics and wood anatomy	Stem theoretical hydraulic conductivity (*K*_s_)	Kg m^−1^ s^−1^ MPa^−1^	16	‐	‐	H3
	Leaf hydraulic conductivity(K_l_)	Kg m^−1^ s^−1^ MPa^−1^	16	‐	‐	H3
	Huber value (HV)	cm^2^ m^−2^	16	66	37	H1, H2, H3
	Conduit diameter (D)	μm	16	‐	11	H3
	Conduit density (CD)	# mm^−2^	16	‐	11	H3
	Lumen fraction (LF)	# μm^−2^	16	‐	‐	H3
Wood and branch traits	Specific branch length(SBL)	cm g^−1^	35	66	37	H1, H2, H3
	Wood density (WD)	g cm^−3^	35	66	37	H1, H2, H3
	Branch water content (BWC)	g g‐1	16	‐	19	H1

Missing values reflect traits that were not measured in some plots. For species shared between islands and the mainland, some missing mainland traits, including additional tree height measurements, were supplemented using data outside plots.

The six traits related to wood anatomy and hydraulics included conduit diameter (D, μm), conduit density (CD, # mm^−2^), lumen fraction (LF, μm^2^ μm^−2^), xylem hydraulic conductivity (*K*_s_, kg m MPa^−1^ s^−1^), leaf hydraulic conductivity (K_l_, kg m MPa^−1^ s^−1^), and Huber value (HV, cm^2^ sapwood area m^−2^ leaf area). D, CD, LF, *K*_s_, and K_l_ are all indicators of hydraulic efficiency and safety. HV indicates allocation to stem and leaf tissues (Tyree & Ewers, 1991), reflecting water‐use strategy: High values indicate greater water‐conducting area per unit transpiring area, which can reduce xylem tension and contribute to hydraulic safety in dry environments (Mencuccini *et al*., 2019; Flo *et al*., 2021). To reduce ontogenetic variation, we sampled 2‐ to 4‐cm‐long branches from 2‐yr‐old branches and stored them in 75% ethanol for measuring wood anatomy (Fig. S2). We used a sliding microtome (Leica, SM2010R) to cut the cross section with a thickness of *c*. 15–30 μm. Then, we stained the sections with safranin for 10–15 min to improve tissue differentiation (lignified tissues acquire a red color). Afterward, we washed the sections with distilled water, and 50, 75, 96, and 100% ethanol (Song *et al*., 2021, 2023). Finally, we fixed the sample in distilled water and took pictures of cross sections of earlywood at ×10 or ×20 magnification with a Leica DM6B microscope and a high‐resolution camera (INFINITY 5C NS, Infinity Software v.6.5.4). After taking the pictures, we measured conduit size, conduit density, and LF using Image Pro Plus 7.0C. LF refers to the ratio of total lumen area to the corresponding total xylem area. Since earlywood determines xylem conductivity and embolism resistance to a larger extent than latewood (Song *et al*., 2021), we randomly selected 50–100 conduits per individual in the earlywood to measure conduit diameter (D, μm), conduit density (CD, # mm^−2^), and conduit LF (μm^2^ μm^−2^). Then, we calculated stem hydraulic conductivity (*K*_s_, kg m MPa^−1^ s^−1^) based on the theoretical Hagen–Poiseuille law (Sterck *et al*., 2008):

$$K_{s}=\left(\pi \rho /128\eta \right)\times \mathrm{CD}\times D^{4}$$

where *ρ* and *ƞ* are water density (998.2 kg m^−3^) and water viscosity (1.002 × $10^{-3}\;\mathrm{Pa}\;s$) at 20°C, respectively. The large range in tropical broadleaf *K*_s_ is an expected consequence of the D^4^ scaling, rather than an inconsistency in measurement or computation. To measure HV, we collected 1‐yr‐old branches. HV was calculated as sapwood area divided by the attached leaf area. High HV reduces leaf‐level transpiration relative to supply and thus is considered a drought tolerance mechanism (van der Sande *et al*., 2019). Leaf hydraulic conductivity (K_l_, kg m MPa^−1^ s^−1^) was calculated as K_l_ = HV × *K*_s_ (Tyree & Ewers, 1991).

The three traits related to branch size and density were SBL (cm g^−1^), branch water content (BWC, g g^−1^), and WD (g cm^−3^). To reduce phenotypic variations, we sampled the standardized 1‐yr‐old branches to measure SBL (Fig. S2). SBL indicates biomass efficiency for branch expansion, which was calculated as the branch length divided by its dry mass (Poorter *et al*., 2018). To measure BWC and density, we selected a 5‐cm‐long branch (Fig. S2). BWC was calculated as the weight difference (i.e. fresh weight‐dry weight) over fresh weight. WD was calculated as dry mass per unit fresh volume.

We collected the 13 traits mentioned previously (Table 1) on 16 of 35 islands. In both mainland and island plots, we always collected seven traits (i.e. H, MLA, SLA, LDMC, HV, WD, and SBL) per species per plot due to time limitations. These seven traits were used to further assess trait associations at species and community levels in both the island and mainland. At the species level, we obtained H measurements for 50 species shared across mainland and island sites, since H was also measured for many species occurring outside the plot boundaries. For the remaining traits, we identified 37 shared species for MLA, SLA, LDMC, HV, SBL, and WD; 19 shared species for BWC; 11 shared species for D and CD; and none for LF, *K*_s_, and K_l_. Therefore, tested the island rule using 10 of 13 traits along eastern mainland‐island transitions.

### Analyses

To compare environmental conditions between island and mainland sites, we applied the nonparametric Mann–Whitney *U*‐test. Analyses were conducted separately for climatic variables (wind speed, light availability, aridity index, minimum and maximum temperature, and vapor pressure deficit; Methods S1) and soil properties (soil carbon, nitrogen, phosphorus, and water content; Methods S2). To test the island rule, we conducted a linear regression of natural‐log‐transformed trait values of the island‐to‐mainland‐ratio (log_e_ RR) against natural‐log‐transformed mainland trait values. A negative relationship indicates that large trait values on the mainland are associated with smaller values on islands (Biddick *et al*., 2019). To test whether the island rule affected species trait associations, we first applied ordinary least squares regression for pairwise trait relationships based on all 13 traits, thus leading to 78 trait–trait slopes. The ordinary least squares regression was performed using the lmodel2 package (Legendre & Oksanen, 2018). Second, we conducted linear regression using pairwise trait slopes as dependent variables, one of the related log‐transformed trait values (log_e_ RR) as independent variables. To further assess whether soil conditions and biogeography affect species trait associations, we regressed the slopes of trait associations against soil nutrients, soil water content, island area, and remoteness (Methods S1).

To evaluate how traits are associated and to compare trait–trait relationships between the island and the mainland, we performed principal component analysis (PCA) at the species and community levels. Species‐level PCA was based on seven functional traits for 37 species shared between island and mainland sites. To quantify the degree to which shared species exhibited acquisitive or conservative strategies, we used PC1 or PC2 scores derived from mainland data at the species level. We then tested whether conservative species tended to shift toward more acquisitive trait values on islands, and whether acquisitive species tended to shift toward more conservative trait values on islands, by modeling log_e_ RR as the dependent variable and PC scores as the independent variables. Community‐level PCA was based on CWM trait values calculated from all species in each community, weighted by their proportional basal area, reflecting ecological processes and functions such as water transport (Poorter *et al*., 2021). PCAs were performed using the factoextra package (Kassambara & Mundt, 2017).

To further explore how traits were statistically correlated in the mainland and island, we did Pearson correlations at the species and community level, and *P*‐adjusted values were applied (Table S3). Pair‐wise linear regressions were also used to explore trait associations. To explore how all 13 traits, including wood anatomical traits, were correlated, we also performed PCA and Pearson's correlations on 16 of 35 islands with complete datasets (Table S4; Fig. S3). To place trait associations within the global context, we extracted global species WD and *K*_s_ (*n* = 630 species) from Chave *et al*. (2009), global species SLA (*n* = 348) from Maire *et al*. (2015), and Global CWM (*n* = 39 communities) from W. R. Anderegg *et al*. (2018). *K*_s_ was calculated from conduit diameter and conduit density using the same method as at our sites. For all analyses, we log‐, cubic root‐, or square‐root‐transformed trait data when necessary to improve normality and homoscedasticity. All data were analyzed using R v.4.4.0 (R Core Team, 2012).

## Results

### Species‐level trait shifts and trait association under the island rule

**Fig. 2:**
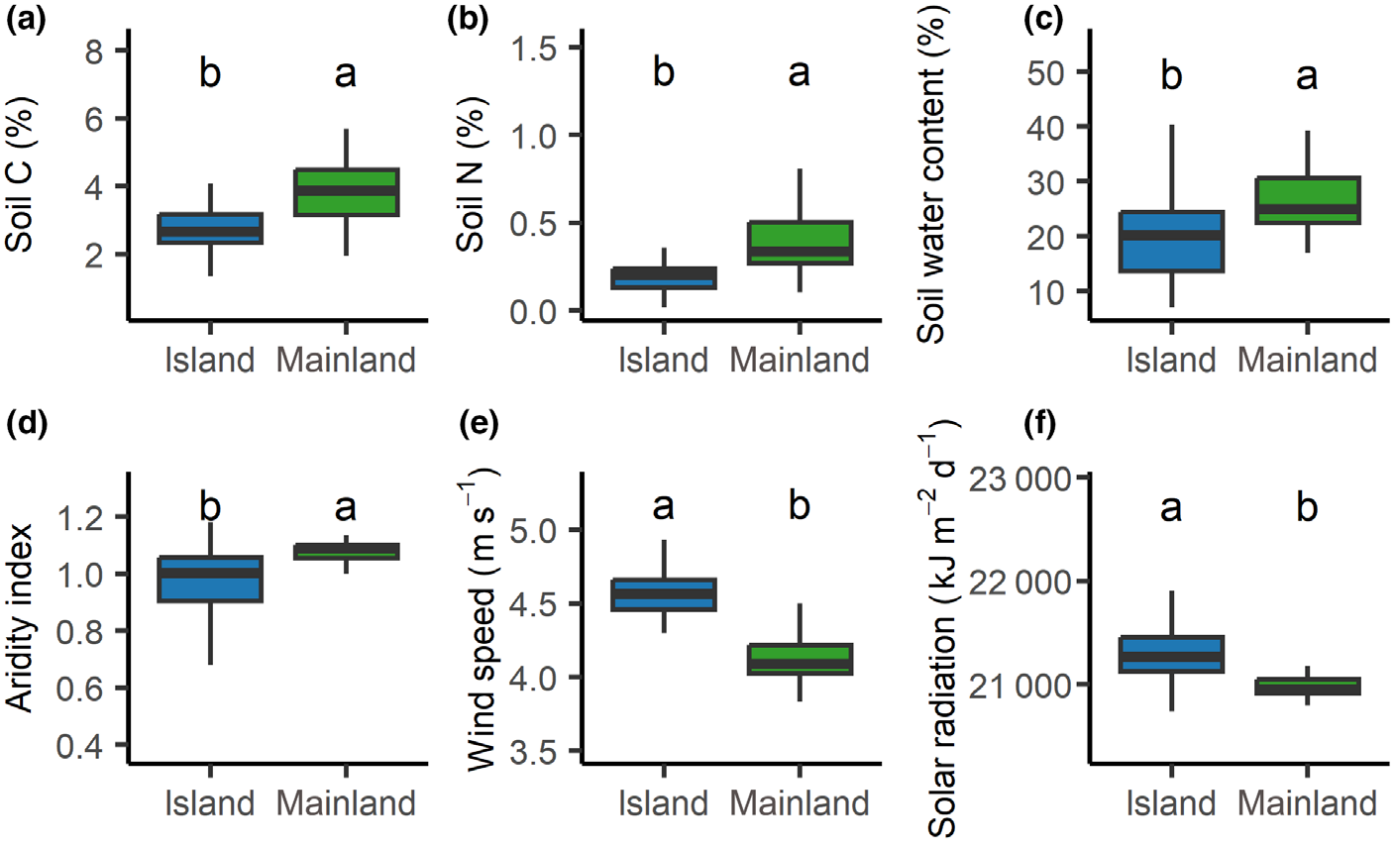
Soil properties and climate on island and mainland plots. (a–c) Show that island soils have lower nutrient availability and are drier than mainland sites. (d–f) Show that island climates have lower water availability, stronger wind, and light conditions. Boxes represent the interquartile range (IQR), horizontal lines indicate medians, and whiskers extend to 1.5 × IQR. Different letters above boxes indicate statistically significant differences at *P* < 0.05 (Wilcoxon rank‐sum test).

**Fig. 3:**
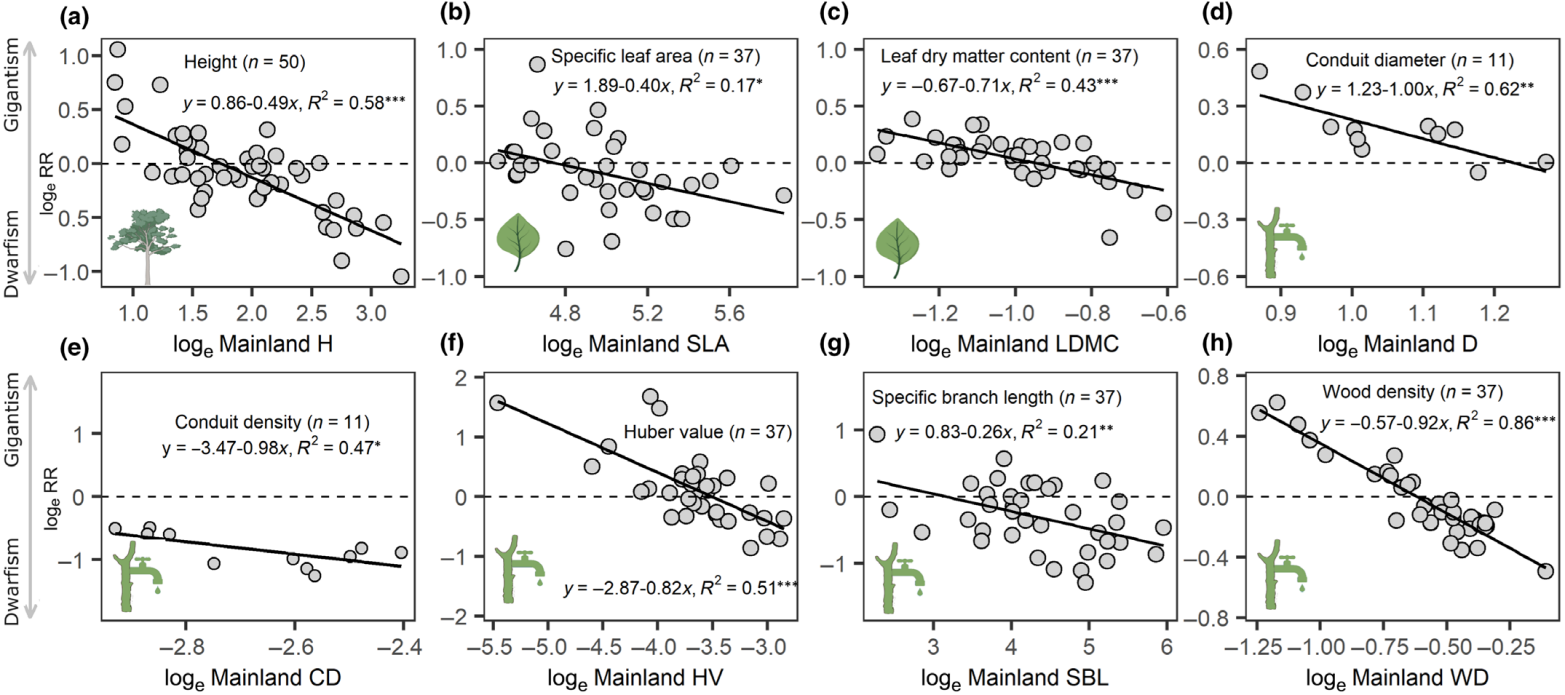
Plant functional trait shifts follow the island rule. Insular size changes (*y*‐axis, log_e_ RR) are regressed against mainland trait values (*x*‐axis). (a) Plant height. (b, c) Leaf traits: specific leaf area (SLA) and leaf dry matter content (LDMC). (d–h) Wood and hydraulic traits: conduit diameter (D), conduit density (CD), Huber value (HV), specific branch length (SBL), and wood density (WD). The dashed horizontal line at zero indicates morphological isometry. Both axes are logarithm‐transformed. Gray points denote single island–mainland pairs. Regression lines, equations, and *R* ^2^ are shown for significant relationships. *, *P* < 0.05; **, *P* < 0.01; ***, *P* < 0.001. Trait icons were obtained from ‘BioRender’ (https://BioRender.com/2t5m5fg).

The island environment was significantly harsher than the mainland, characterized by poorer soil conditions (lower soil carbon and nitrogen; Fig. 2a,b), reduced water availability (lower aridity index and soil water content; Fig. 2c,d), and stronger winds and higher light conditions (Fig. 2e,f). Within this harsher insular environment, we tested the island rule that large functional trait values on the mainland would be smaller on islands and vice versa (Fig. 3). Eight of the 10 traits exhibited significant trends from gigantism with big trait values to dwarfism with small trait values that are consistent with the island rule, specifically H, SLA, LDMC, conduit diameter (D), conduit density (CD), HV, SBL, and WD. Individual MLA and BWC did not show trends (*P* > 0.05).

**Fig. 4:**
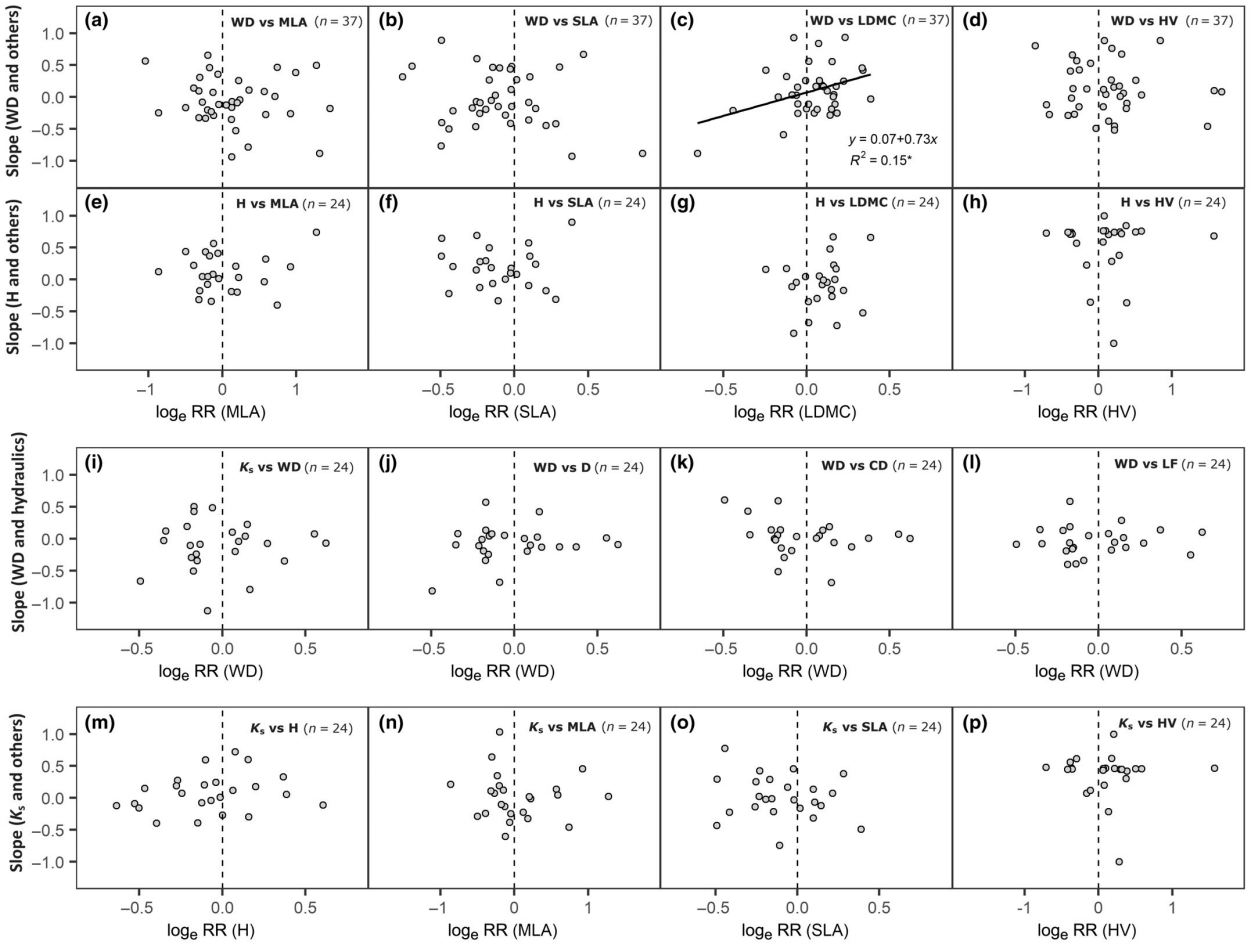
The island rule does not affect species‐level trait associations. The *x*‐axis indicates insular size change (log_e_ RR; *n* = 24 or 37), and the *y*‐axis indicates the related pairwise trait slope. log_e_ RR < 0, log_e_ RR = 0, and log_e_ RR > 0 indicate dwarfism, stasis, and gigantism, respectively. (a–d) Trait associations between wood density (WD) and other traits related to leaf and hydraulics: MLA, leaf area; SLA, specific leaf area; LDMC, leaf dry matter content; HV, Huber value. (e–h) Trait associations between tree height (H) and other traits related to leaf and hydraulics. (i–l) Trait associations between WD and traits related to hydraulics and wood anatomy: *K*_s_, xylem hydraulic conductivity; D, conduit diameter; CD, conduit density; LF, lumen fraction. (m–p) Trait associations between *K*_s_ and traits related to size and hydraulics. Regression lines, equations, and *R* ^2^ are shown for significant relationships. *, *P* < 0.05; **, *P* < 0.01; ***, *P* < 0.001.

Despite significant changes in trait values consistent with the island rule (Fig. 3), no impact of these shifts was found on species‐level trait associations except the WD‐LDMC relationship (Fig. 4). Instead, island area, soil nutrients, and soil water content significantly affected trait associations (Fig. S4). Leaf size‐related trait associations (MLA and SLA) with WD and H both became more positive on islands with large area, and dry and nutrient‐poor (low soil water content and low soil N; Fig. S4a–c,e–g). By contrast, associations between leaf toughness (high LDMC) and WD became negative, while those with H became positive under dry soil conditions (Fig. S4d,h). Relationships between WD and hydraulic efficiency traits (i.e. high D, CF, and high hydraulic conductivity‐*K*_s_) shifted from negative to positive on larger islands and wet soil (Fig. S4i–k). Positive associations between *K*_s_ and HV weakened under dry, nutrient‐poor soil conditions (Fig. S4l–n). Associations between *K*_s_ and leaf size (MLA) shifted from negative to positive in nutrient‐poor soil (Fig. S4o,p).

### Traits associations at species and community levels

**Fig. 5:**
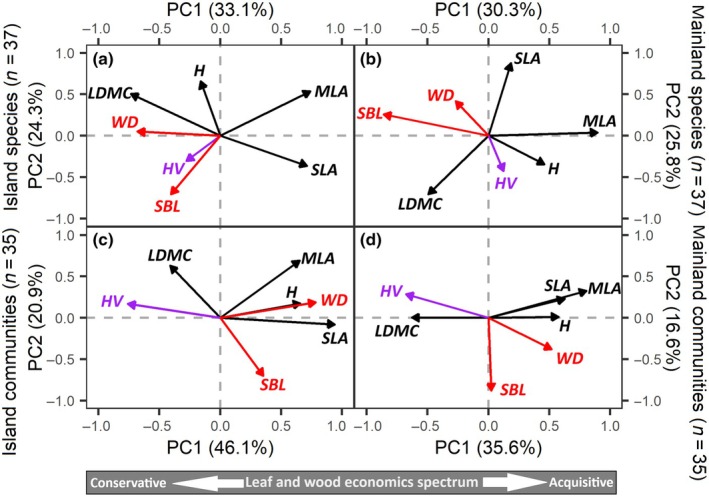
Principal components analysis (PCA) of functional traits for island and mainland plants. The top (a, b) and bottom (c, d) rows show species and CWM levels, respectively, along the first two PCA axes. Seven traits related to leaf and plant height (black), hydraulic (HV in purple), and branch size and density (red). H, tree height; HV, Huber value; LDMC, leaf dry matter content; MLA, leaf area; SBL, specific branch length; SLA, specific leaf area; WD, wood density.

Island species followed a leaf‐wood economics spectrum, running from mechanical safety (large WD and LDMC) to the left and high carbon assimilation with strong light capture capacity (MLA and SLA) to the right along the first axis of PCA (Fig. 5a). This leaf‐wood economics spectrum was weaker for mainland species since WD is largely orthogonal to the SLA‐LDMC axis (Fig. 5b). PC scores of mainland species (Fig. 5b) indicated that conservative species shifted toward more acquisitive and higher values of H, SLA, and BWC on islands, while acquisitive species shifted toward more conservative and higher values of LDMC and WD on islands, while acquisitive species had more conservative and larger values in LDMC and WD on islands (Fig. S5). Together, these patterns indicate that functional trait shifts were consistent with the island rule.

Island and mainland CWM traits followed a similar ‘hydraulic‐wood‐plant size’ economics spectrum, characterized by coordinated variation among hydraulic traits (HV), wood traits (WD), and size‐related traits (SLA and MLA). This hydraulic‐wood‐plant‐size spectrum runs from leaf and hydraulic conservative traits (i.e. large HV and LDMC) on the left, to size‐related acquisitive traits (large H, MLA, SLA) and WD on the right (Fig. 5c,d). By contrast, WD and SLA exhibited a trade‐off at the island species level but clustered in the same direction at the island community level (Fig. 5a,c).

**Fig. 6:**
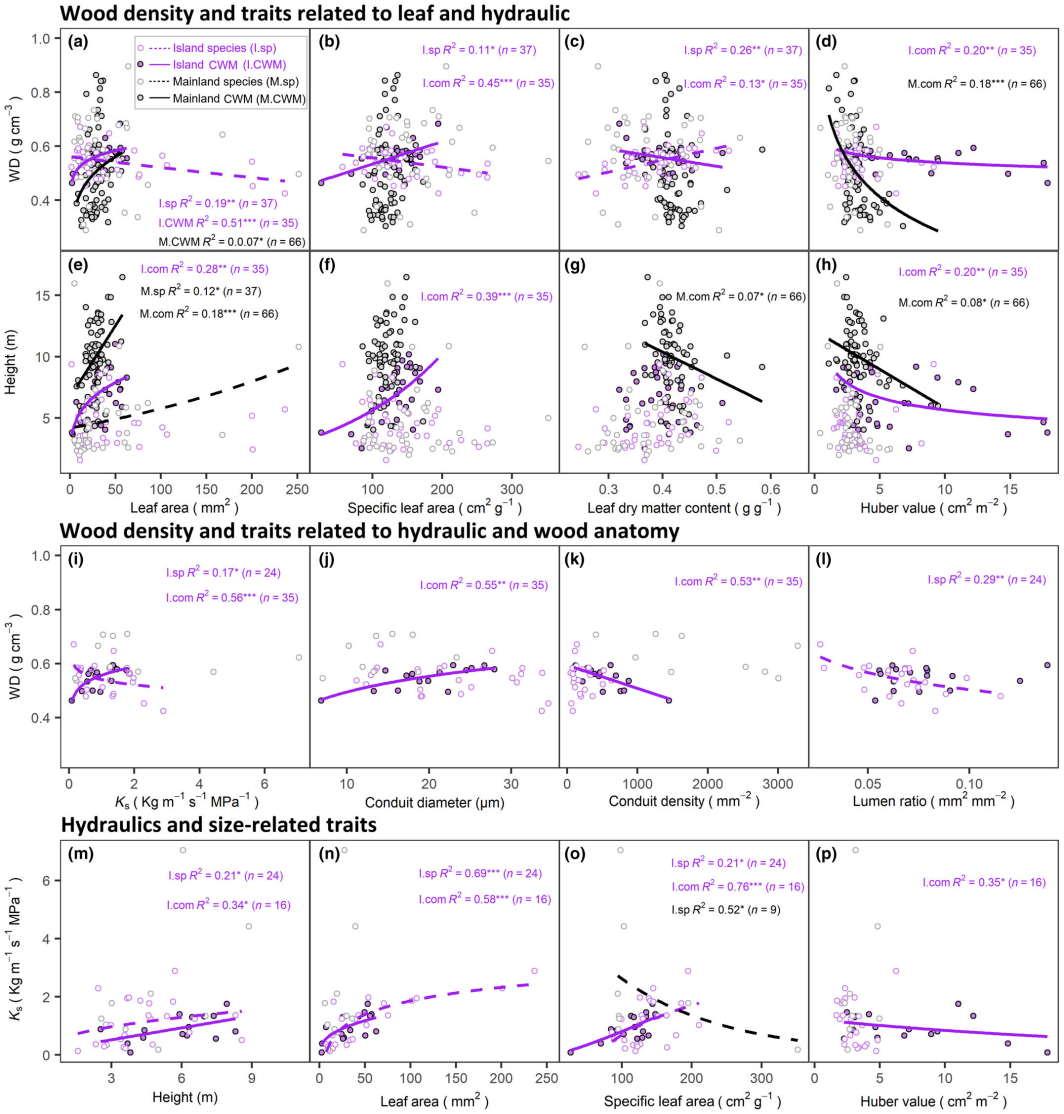
Bivariate species‐level and community‐weighted mean (CWM) trait associations for island and mainland plants. (a–d) Relationships between wood density (WD) and traits related to leaf and hydraulics. (e–h) Relationships between tree height and traits related to leaf and hydraulics. (i–l) Trait relationships between WD and traits related to hydraulics and wood anatomy. (m–p) Trait associations between hydraulics and size‐related traits. K_s_, xylem hydraulic conductivity. Purple colors with closed circles denote island community level (I.CWM) and open circles denote species level (I.sp). Gray colors with closed circles denote mainland community level (M.CWM) and open circles denote species level (M.sp). Purple solid and dashed lines indicate regressions at the island community level (*n* = 16 or 35) and species level (*n* = 24 or 37), respectively. Black solid and dashed lines indicate regressions at the mainland community level (*n* = 66) and species level (*n* = 9 or 37), respectively. *R* ^2^ and significant regression lines are shown. Data were log‐transformed when necessary. Regression equations are provided in Supporting Information Table S5. *, *P* < 0.05; **, *P* < 0.01; ***, *P* < 0.001.

When directly comparing island plants at the species and community levels, we found surprisingly contrasting trait associations between WD and traits related to leaf and wood anatomy, and consistent relationships between *K*_s_ and size‐related traits (Fig. 6; Tables S3‐S5). Specifically, island species WD was negatively correlated with MLA, SLA, *K*_s_, K_l_, and LF (Fig. 6a,b,i,l), and positively with LDMC (Fig. 6c). By contrast, island CWM WD was positively correlated with CWM MLA, SLA, *K*_s_, and conduit diameter (Fig. 6a,b,i,j); and negatively correlated with LDMC, HV, and conduit density (Fig. 6c,d,k). At both island species and community levels, *K*_s_ was always positively related to H, MLA, and SLA (Fig. 6m–o). Across both species and community levels, trait associations exhibited broadly consistent patterns between island and mainland plants. For both island and mainland plants, CWM WD was negatively correlated with CWM HV (Fig. 6d); CWM H was always positively associated with CWM MLA (Fig. 6e), and negatively associated with CWM HV (Fig. 6h); SLA was always negatively correlated with LDMC at both scales (Table S5).

## Discussion

We investigated 13 plant functional traits related to hydraulics and carbon use across 35 island communities and 66 nearby mainland communities in eastern China, including 37 shared species, to test whether trait values and trait associations were influenced by the island rule at the species level, and whether species‐ and community‐level trait associations diverged along the conservative‐acquisitive spectrum. Consistent with the island rule, we found shifts in several functional traits related to size, wood anatomy, and hydraulics (Fig. 3). However, these shifts did not alter species‐level trait associations (Fig. 4). While trait associations were generally conserved between island and mainland species, we observed contrasting patterns between species‐ and community‐level trait associations on islands (Figs 5, 6). Below, we discuss the ecological mechanisms and implications of these patterns across organizational scales in insular systems.

### The island rule in structural, hydraulic, and carbon traits

As expected, we observed the island rule in functional traits related to size (height), hydraulics (HV and wood anatomy), and leaf carbon economic traits (SLA and LDMC; Fig. 3). Acquisitive species on mainland shifted toward more conservative directions of H, SLA, LDMC, and WD on island, whereas conservative mainland species shifted toward relatively more acquisitive directions on island (Fig. S5), indicating convergence in trait shifts under insular conditions. Although increased stature can enhance light capture and photosynthetic capacity, it also imposes greater hydraulic constraints, increasing vulnerability to hydraulic failure and carbon starvation (Fernández‐de‐Uña *et al*., 2023). Consequently, the‐island‐rule–mediated shifts in height may alter how island species balance water transport safety and carbon gain, with implications for ecosystem‐level water and carbon fluxes under future drier climates. However, leaf size did not conform to the island rule (*P* > 0.05), a pattern broadly consistent with Cox & Burns (2017) and Burns (2019) but not with Biddick *et al*. (2019). This inconsistency likely reflects the high phenotypic plasticity of leaf size in response to local biotic (e.g. competitors and predators) and abiotic factors (e.g. light, water, and nutrients), potentially intensified by diverse herbivore communities on these islands (Zhang *et al*., 2025). Notably, island species exhibited lower variances in WD, *K*_s_, and CD than mainland species (Table S6), indicating convergence in hydraulically integrated xylem traits toward a narrow insular structural optimum under directional selection. Together, this convergence suggests strong selection on hydraulic trait coordination under limited water availability in insular environments.

From the ecological perspective, trait shifts likely reflect coordinated responses to multiple selective pressures, including convergent evolution, climate factors, soil properties, phenotypic plasticity, competitors, and predators (Meiri *et al*., 2008; Biddick *et al*., 2019; Benítez‐López *et al*., 2021). Our previous work found stronger dwarfism in plant height on resource‐poor and plant‐competitive islands (Zhang *et al*., 2025), highlighting the role of ecological filtering in shaping size shifts. The patterns of dwarfism under poor habitats align with broader ecological adaptations to environmental stress rather than an island‐specific response (King, 1990). Island biogeography was expected to affect trait shifts since it was linked to resource availability (Xu *et al*., 2023). Trait values varied with island area and remoteness (Fig. S6). However, neither island area nor remoteness affected trait shifts (log_e_ RR) (Methods S2). The climate variables weakly affected log_e_ RR (Fig. S7). Climatic variables showed weak effects on log_e_ RR (Fig. S7). These results likely suggest that trait variation among shared species is shaped more strongly by evolutionary processes than by geographic isolation *per se*. While we acknowledge that plant traits can be influenced by fine‐scale microhabitat conditions (De Pauw *et al*., 2022), such microhabitat data were not available in our study. Future studies should integrate microhabitat variation and evolutionary processes to better explore the mechanisms underlying trait shifts in island plants.

### Species‐level trait associations are conserved despite the island rule

Although we expected that trait value shifts might modify species‐level trait associations, we found these associations remained largely stable. It implies that island plants can maintain coordinated growth and survival strategies with biophysical and evolutionary constraints. According to the Equilibrium Theory of Island Biogeography, functionally similar species may replace one another during community turnover, preserving trait associations with the community equilibrium despite compositional change (MacArthur & Wilson, 1963). Stable species‐level trait associations may therefore reflect consistent trait filtering during community assembly, supported by previous evidence of temporal stability in functional trait values (Schrader *et al*., 2023). Evolutionary convergence among traits (Biddick *et al*., 2019), such as allometric relationships among different organ sizes (Corner, 1949), may enable plants to adjust to shifts in resource availability through similar functional strategies, potentially contributing to the stability of species‐level trait associations. Although we could not directly test the island rule's impact on CWM trait associations, we expect that filtering by island environments results in communities composed of species with similar functional traits, leading to stability in CWM trait patterns as well.

Species‐level trait associations shift across soil conditions, reflecting context‐dependent coordination among plant functional traits. Positive associations between leaf acquisitive traits (high MLA and SLA) and both WD and H in dry and nutrient‐poor soils (Fig. S4b,c,f,g) suggest tighter integration of leaf and wood structures to maintain light capture, water transport, and mechanical support under resource limitation (Wright *et al*., 2004; Díaz *et al*., 2016). Associations between WD and hydraulic efficiency traits shifted from negative to positive under wet conditions (Fig. S4k) point to a relaxation of the safety–efficiency trade‐off, enabling co‐optimization of conductivity and mechanical strength (Gleason *et al*., 2016). In dry, nutrient‐poor soils, only trees with high hydraulic conductivity can support large leaves, leading to a positive *K*_s_‐MLA association (Fig. S4o,p) as an adaptive coordination under stress (Manzoni *et al*., 2013). Together, these findings highlight how environmental filtering alters trait associations and suggest flexible trait coordination strategies in response to island‐specific abiotic conditions.

### Island plant trait associations in the context of the global leaf and wood economics spectrum

We found a consistent wood‐leaf economic spectrum across island species under global trait economics frameworks. At the species level, wood and leaf traits varied across a coupled conservative–acquisitive gradient: from conservative strategies emphasizing hydraulic safety and tissue toughness (e.g. high WD and HV), to acquisitive strategies with enhanced hydraulic and carbon assimilation capacity (e.g. high *K*_s_, K_l_, SLA, MLA; Figs 5, S3). These spectra reflect trade‐offs between hydraulic and mechanical safety vs hydraulic efficiency, and between leaf construction costs and growth potential. The global plant economics spectrum (Reich, 2014) posits that plants integrate morphological, anatomical, and physiological traits across organs to adopt either an acquisitive strategy (i.e. fast growth, low tissue density, short lifespan, and high resource uptake) or a conservative strategy (i.e. slow growth, dense tissues, and long lifespan). While some studies found decoupling of leaf and wood economic traits in tropical and subtropical forests (Markesteijn *et al*., 2011; Li *et al*., 2015), others found coupled relationships in temperate forests (Song *et al*., 2022) or at global scales (Mencuccini *et al*., 2019). Our results support a coupled spectrum across both temperate mainland and island plants.

At the community level, we also observed trait spectra consistent with species‐level trait coordination. Acquisitive communities (e.g. high H, SLA, MLA) exhibit high water and carbon flux potential, while conservative communities are characterized by greater hydraulic safety (e.g. high HV) and tissue density (e.g. high LDMC). Larger plant organs (i.e. height and leaf size) facilitate greater light capture and water transport capacity, supporting higher carbon gain and biomass production (Liu *et al*., 2019; Song *et al*., 2022). These findings confirm that economic spectra scale from species to communities, including in insular ecosystems. Such coordinated strategies are critical for improving ecosystem modeling. Coupled trait spectra can inform the parameterization of hydrological models, Dynamic Global Vegetation Models (DGVMs), and Earth System Models (ESMs), especially for predicting carbon fluxes, plant fitness, and ecosystem resilience under climate change.

### Diverging species–community scaling in island trait associations

Some community‐level trait associations on islands aligned with species‐level patterns and were even stronger, whereas others diverged markedly. Specifically, island CWM *K*_s_ was positively related to larger leaf and plant size (MLA, SLA, and H; Fig. 6i–k), and negatively related to hydraulic safety (HV; Fig. 6l), reflecting a strategy of high water and carbon flux at the cost of hydraulic safety. Similarly, island CWM H was positively associated with MLA, SLA, *K*_s_, D, and negatively with HV (Tables S3, S4), suggesting that taller island communities could grow fast with strong carbon assimilation capacity with large leaves and high hydraulic efficiency through big conduit size, but at the cost of reduced hydraulic safety with low HV. Therefore, taller communities would be more vulnerable to more severe and frequent drought events in a drier future.

**Fig. 7:**
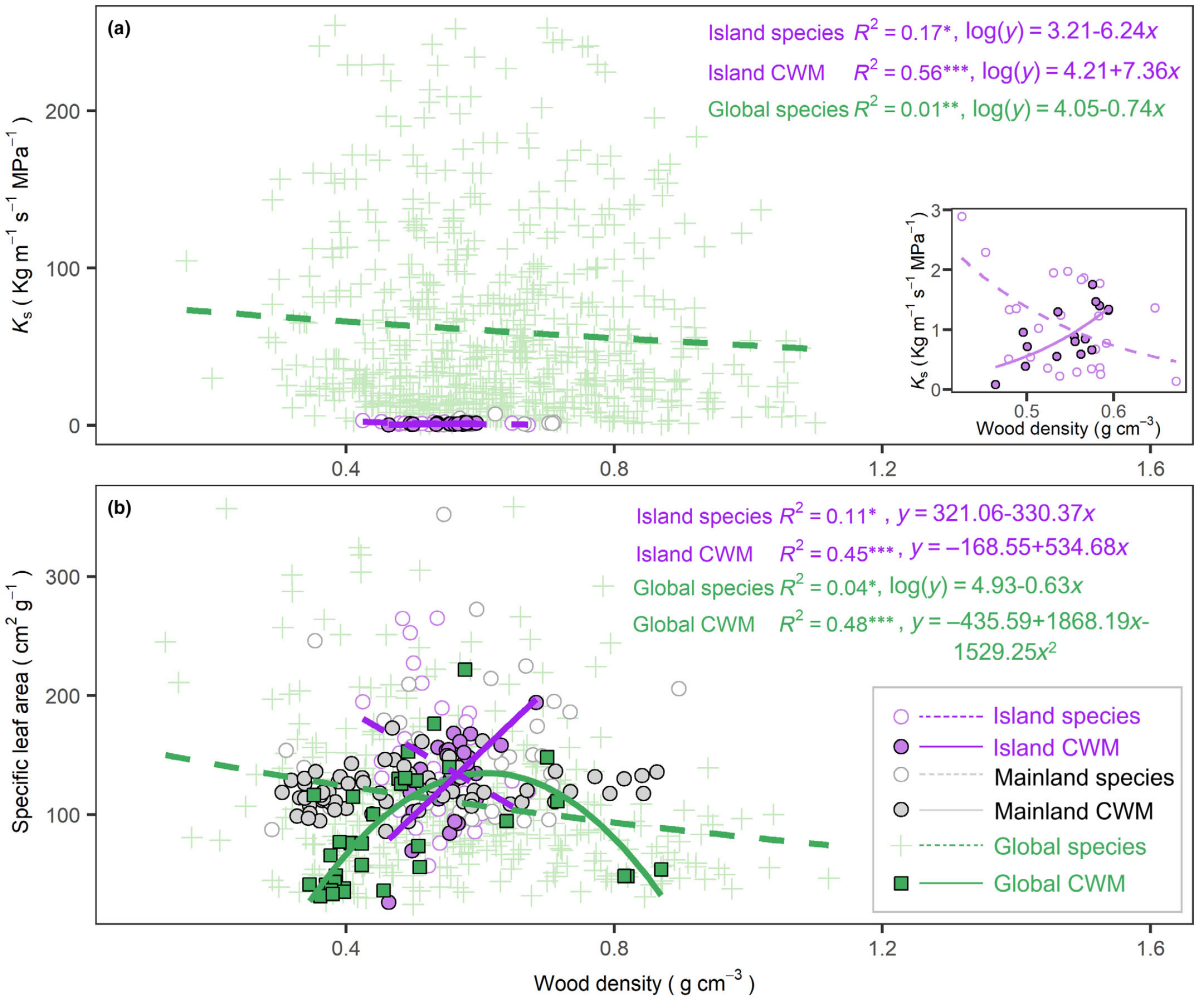
Wood density and acquisitive traits related to hydraulic and leaf economics at species and community levels. (a) Global relationships between wood density and hydraulic conductivity (*K*_s_): significant relationships from the island data are magnified in the bottom‐right inset. (b) Global relationships between wood density and specific leaf area. Solid and dashed lines indicate significant and nonsignificant regressions, respectively. Global species wood density and hydraulic conductivity (*n* = 630 species) were obtained from Chave *et al*. (2009). Global species‐specific leaf area (*n* = 348) was downloaded from Maire *et al*. (2015). Global community‐weighted mean (CWM) (*n* = 39 communities) was retrieved from W. R. Anderegg *et al*. (2018). Regressions were fit on log‐transformed data when applicable. *R* ^2^, significant regression lines, and fitted equations are shown. *, *P* < 0.05; **, *P* < 0.01; ***, *P* < 0.001.

However, we found a striking contrast in the scaling relationships of WD. At the species level, WD was negatively associated with hydraulic efficiency traits (*K*_s_, K_l_, and LF; Table S4) and carbon acquisitive traits (carbon assimilation efficiency: MLA and SLA; Fig. S4b,c), consistent with global datasets (Fig. 7a,b). Yet at the community level, CWM WD positively correlated with acquisitive traits such as large leaf size (i.e. MLA, SLA; Fig. 6a,b) and high hydraulic efficiency (i.e. *K*_s_, and D; Fig. 6i–j). This unexpected reversal may reflect the disproportionate influence of dominant species with dense wood and acquisitive traits, an adaptation to windy or mechanically demanding island environments.

Global datasets also suggest a nonlinear relationship: CWM, WD, and SLA are positively correlated when WD < 0.6 g cm^−3^ but negatively correlated at higher WD values (Fig. 7b). WD was generally expected to be negatively related to D, *K*_s_, MLA, and SLA, because tissue density is largely determined by the space dedicated to conduits, and high transpiration through big leaves enables high water transport via large conduits (Chave *et al*., 2009). This may be explained by anatomical differences, such as the contribution of nonconductive fibers to WD (Hacke *et al*., 2001), or by a decoupling between conduit allocation and mechanical support.

Moreover, ecosystem‐scale trade‐offs can diverge from species‐level patterns due to the influence of dominant species. While strong conservative–acquisitive trade‐offs exist at the species level, they may weaken or disappear at the community or ecosystem levels (L. D. Anderegg *et al*., 2018; Anderegg *et al*., 2024), particularly under habitat filtering or niche differentiation (Ackerly & Cornwell, 2007). Despite similar species richness across island and mainland sites (27 ± 3 vs 25 ± 1 species), dominant species ultimately shape community‐level trait patterns and functional composition more than species richness or trait range alone.

These positive associations between CWM WD and acquisitive traits among island communities suggest that woody plant communities can adopt an acquisitive strategy while maintaining dense wood, which provides mechanical support and reduces the risk of stem breakage under strong winds. WD is the nexus linking many plant functions, including water storage, hydraulic conductivity, carbon assimilation, drought tolerance, mechanical resistance, nutrient retention, growth, and survival (Poorter *et al*., 2019). This underscores the importance of WD in structuring community functional composition and influencing ecosystem functioning, particularly in island plant communities. To our knowledge, this is the first study to report a contrasting scaling relationship between WD and carbon–hydraulic acquisitive traits across species and community levels. It provides a foundation for improving island ecosystem models of carbon and water transport, particularly given the central role of water transport in these systems (Li *et al*., 2021; Anderegg *et al*., 2024). Overall, our study highlights how insular plant communities offer a valuable system for scaling up trait–function relationships to refine global models of ecosystem structure and dynamics.

### Implications for science and conservation

Our study highlights the importance of island rule and trait economics frameworks for understanding plant adaptation, ecosystem function, and community assembly. First, species‐level relationships align with global expectations, while community‐level patterns diverge, possibly due to the influence of dominant species with dense wood and fast‐growth traits, indicating the island community trade‐offs beyond the global spectrum space, whereas the leaf and wood economics spectrum remains within the global WES and LES. Second, our results guide island plant conservation and vegetation restoration in a drier climate future. Trait‐based criteria, such as combining high WD with large SLA and hydraulic efficiency, can inform community selection for restoration. In windy, humid environments, dense‐wooded, acquisitive species may offer both mechanical resilience and high productivity. In dry, exposed habitats, conservative strategies with smaller leaves and higher hydraulic safety are suggested. Ultimately, our findings emphasize the need to incorporate scale‐dependent trait dynamics into predictive models. Accounting for community processes and dominant functional types can improve forecasts of ecosystem function under climate change, especially in island ecosystems.

To conclude, our study extends the island rule to functional traits related to plant carbon and hydraulic economics, revealing convergent size optimization strategies for resource‐use efficiency in resource‐limited environments. Island and mainland plants exhibit global WES and LES at both species and community levels, highlighting their generality in shaping forest function under insular conditions and improving the forest dynamics and Earth System Models in predicting plant responses to climate change. Nevertheless, the species‐level WD and the acquisitive leaf‐hydraulic trade‐offs disappear but are reversed into positive associations at the community level. This counterintuitive pattern was largely driven by dominant species with integrated traits that favored rapid growth and high hydraulic efficiency without compromising mechanical strength, suggesting that species composition, rather than the island rule, can restructure trait associations at the community scale, ultimately influencing carbon sequestration and ecosystem function. Our study highlights the importance of integrating trait‐based theory and modeling with conservation and restoration strategies to safeguard island ecosystems in a rapidly changing climate.

## Competing interests

None declared.

## Author contributions

YS, NGM, EY and SD conceived the idea and contributed to the study design and interpretation of results. YS performed the analyses and wrote the first draft of the manuscript. ZZ, WR, MX and DH contributed to data collection and analyses. ALP contributed to writing. All authors contributed substantially to revisions.

## Disclaimer

The New Phytologist Foundation remains neutral with regard to jurisdictional claims in maps and in any institutional affiliations.

## Supporting information


**Fig. S1** Overview of the study area on the 35 eastern islands of China and the nearby 66 mainland plots.
**Fig. S2** Trait collection from each branch.
**Fig. S3** Principal components analysis for the first two axes based on all 13 traits for plants in 16 islands at different organization levels.
**Fig. S4** Island area and soil properties affect species‐level trait associations.
**Fig. S5** The conservative‐acquisitive strategies follow the island rule.
**Fig. S6** The significant impacts of island area, island remoteness, and soil nutrients on island CWM traits (*n* = 10–34).
**Fig. S7** Climate data weakly affect species‐level trait shifts.
**Methods S1** Environment data collection.
**Methods S2** Analyses of soil and biogeographic influences on trait shifts.
**Table S1** The geographic information of 35 studied islands in eastern China.
**Table S2** 37 shared species studied in both the island and mainland sites.
**Table S3** Bivariate Pearson correlations among seven studied traits in islands and mainland at species (*n* = 37 in gray) and community level (*n* = 35 or 66 in green).
**Table S4** Bivariate Pearson correlations among 13 complete traits in 16 islands at individual (*n* = 1143), species (*n* = 60), and community level (*n* = 16).
**Table S5** Fitted equations and corresponding formulas for each significant regression line in Fig. 6.
**Table S6** Results of median‐centered Levene's tests comparing trait variance between island and mainland species.
Please note: Wiley is not responsible for the content or functionality of any Supporting Information supplied by the authors. Any queries (other than missing material) should be directed to the *New Phytologist* Central Office.

## Data Availability

Data and code have been deposited in Figshare: doi: 10.6084/m9.figshare.28017491.
