# Supplementary material for: Island influences on plant functional traits and trait–trait associations across species‐ and community‐scales
Source: New Phytol. 2026 Feb 27;250(4):2176–89. doi: 10.1111/nph.71040 (PMC13103414; doi:10.1111/nph.71040)
Supplement: Supplementary file 1 — Fig. S1 Overview of the study area on the 35 eastern islands of China and the nearby 66 mainland plots. Fig. S2 Trait collection from each branch. Fig. S3 Principal components analysis for the first two axes based on all 13 traits for plants in 16 islands at different organization levels. Fig. S4 Island area and soil properties affect species‐level trait associations. Fig. S5 The conservative‐acquisitive strategies follow the island rule. Fig. S6 The significant impacts of island area, island remoteness, and soil nutrients on island CWM traits (n = 10–34). Fig. S7 Climate data weakly affect species‐level trait shifts. Methods S1 Environment data collection. Methods S2 Analyses of soil and biogeographic influences on trait shifts. Table S1 The geographic information of 35 studied islands in eastern China. Table S2 37 shared species studied in both the island and mainland sites. Table S3 Bivariate Pearson correlations among seven studied traits in islands and mainland at species (n = 37 in gray) and community level (n = 35 or 66 in green). Table S4 Bivariate Pearson correlations among 13 complete traits in 16 islands at individual (n = 1143), species (n = 60), and community level (n = 16). Table S5 Fitted equations and corresponding formulas for each significant regression line in Fig. 6. Table S6 Results of median‐centered Levene's tests comparing trait variance between island and mainland species. Please note: Wiley is not responsible for the content or functionality of any Supporting Information supplied by the authors. Any queries (other than missing material) should be directed to the New Phytologist Central Office. [file NPH-250-2176-s001.docx]

## *New Phytologist* Supporting Information

Article title: Island influences on plant functional traits and trait-trait association across species- and community-scales

Authors: Yanjun Song, Nate G. McDowell, Zengke Zhang, Alexandria L. Pivovaroff, Mingshan Xu, Wentao Ren, Dong He, En-Rong Yan, Sylvain Delzon

Article acceptance date: 05 January 2026

The following Supporting Information is available for this article:

**Fig. S1** Overview of the study area on the 35 eastern islands of China and the nearby 66 mainland plots. The islands cover over 12 degrees of latitude. It is a long transect (300 km from west to east, and 1600 km from south to north) across a gradient of humidity (annual aridity index ranges from 0.38 to 1.16).

**Fig. S2** Trait collection from each branch. For trait abbreviations, see Table 1. K_s_ and K_l_ are not shown because they are calculated from wood anatomy and leaf traits.

**Fig. S3** Principal components analysis (PCA) for the first two axes (a-c) based on all 13 traits for plants in 16 islands at different organization levels. Individual-level analyses are shown in the left column (n=1143), species-level analyses are shown in the middle column (n=60), and community-level analyses (n=16) are shown in the right column. Black color indicates traits related to leaf and plant size, and leaf toughness, purple indicates wood anatomy and hydraulics and red indicates branch size and density. For trait abbreviations, see Table 1.

**Fig. S4** Island area and soil properties affect species-level trait associations. The y-axis indicates the related pair-wise trait slope (shown inside each panel). These pair-wise trait slopes correspond to the same trait categories in Fig. 3. WD, wood density; H, tree height; K_s_, xylem hydraulic conductivity; MLA, leaf area; SLA, specific leaf area; LDMC, leaf dry matter content; HV, Huber value; D, conduit diameter; CD, conduit density; LF, lumen fraction. Non-linear regressions reflect log-transformed regressions. R^2^ and significant regression lines are shown. *, *P*<0.05; **, *P*<0.01.

**Fig. S5** The conservative-acquisitive strategies follow the island rule. Insular size changes (y-axis, lnRR=log-transformed (island trait value/mainland trait value)) are shown with conservative-acquisitive strategies (x-axis). The species-specific PC scores were obtained from mainland species in Fig. 4b. **a-c**, tree height (H) and leaf traits. SLA, specific leaf area. LDMC, leaf dry matter content. **d-f**, wood traits for BWC (branch water content), SBL (specific leaf length), and WD (wood density). Fewer than 37 shared species are shown due to missing trait measurements. R^2^ and significant regression lines are shown. *, *P*<0.05; **, *P*<0.01.

**Fig. S6** The significant impacts of island area, island remoteness, and soil nutrients on island CWM traits (n=10-34). The first row indicates relationships between island biogeographic and CWM traits, and the second row indicates relationships between soil nutrients and CWM traits. **a-c**, island area and CWM traits for tree height (a), conduit density (CD, b), and huber value (HV, c). **d-g**, island remoteness and CWM traits for height (d), specific leaf area (SLA, e), leaf dry matter content (LDMC, f), and conduit diameter (D, g). **h-k**, soil nitrogen (N) and CWM traits for SLA (h), LDMC (i), HV (j), and specific branch length (SBL, k). **i-n**, soil phosphorus (P) and CWM traits for wood anatomy in terms of conduit number and size, respectively. A reduced number of 16 island communities was shown, since some trait and soil data were not collected for all 35 island communities. Regression lines, equations and R^2^ are shown. *, *P*<0.05; **, *P*<0.01.

**Fig. S7** Climate data weakly affect species-level trait shifts. The y-axis indicates Insular size changes (y-axis, lnRR=log-transformed (island trait value/mainland trait value)), and the x-axis indicates climate variables. Non-linear regressions reflect log-transformed regressions. R^2^ and significant regression lines are shown. *, *P*<0.05; **, *P*<0.01.

**Table S1** The geographic information of 35 studied islands in eastern China.

**Table S2** 37 shared species studied in both the island and mainland sites.

**Table S3** Bivariate Pearson correlations among 7 studied traits in islands and mainland at species (n=37 in grey) and community level (n=35 or 66 in green). Bold coefficients indicate *P*<0.05. Underlined coefficients indicate adjusted *P*<0.05. For trait variables, see Table 1.

**Table S4** Bivariate Pearson correlations among 13 complete traits in 16 islands at individual (n=1143), species (n=60), and community level (n=16). Bold coefficients indicate *P*<0.05. Underlined coefficients indicate adjusted *P*<0.05. For trait variables, see Table 1.

**Table S5** Fitted equations and corresponding formulas for each significant regression line in Fig. 6.

**Table S6** Results of median-centered Levene’s tests comparing trait variance between island and mainland species. P-values indicate significant differences (*P*<0.05). Traits with insufficient variation in one group (e.g. K_s_, CD) could not be tested with Levene’s test.

**Methods S1** Environment data collection

**Methods S2** Analyses of soil and biogeographic influences on trait shifts

**Fig. S1** Overview of the study area on the 35 eastern islands of China and the nearby 66 mainland plots. The islands cover over 12 degrees of latitude. It is a long transect (300 km from west to east, and 1600 km from south to north) across a gradient of humidity (annual aridity index ranges from 0.38 to 1.16).


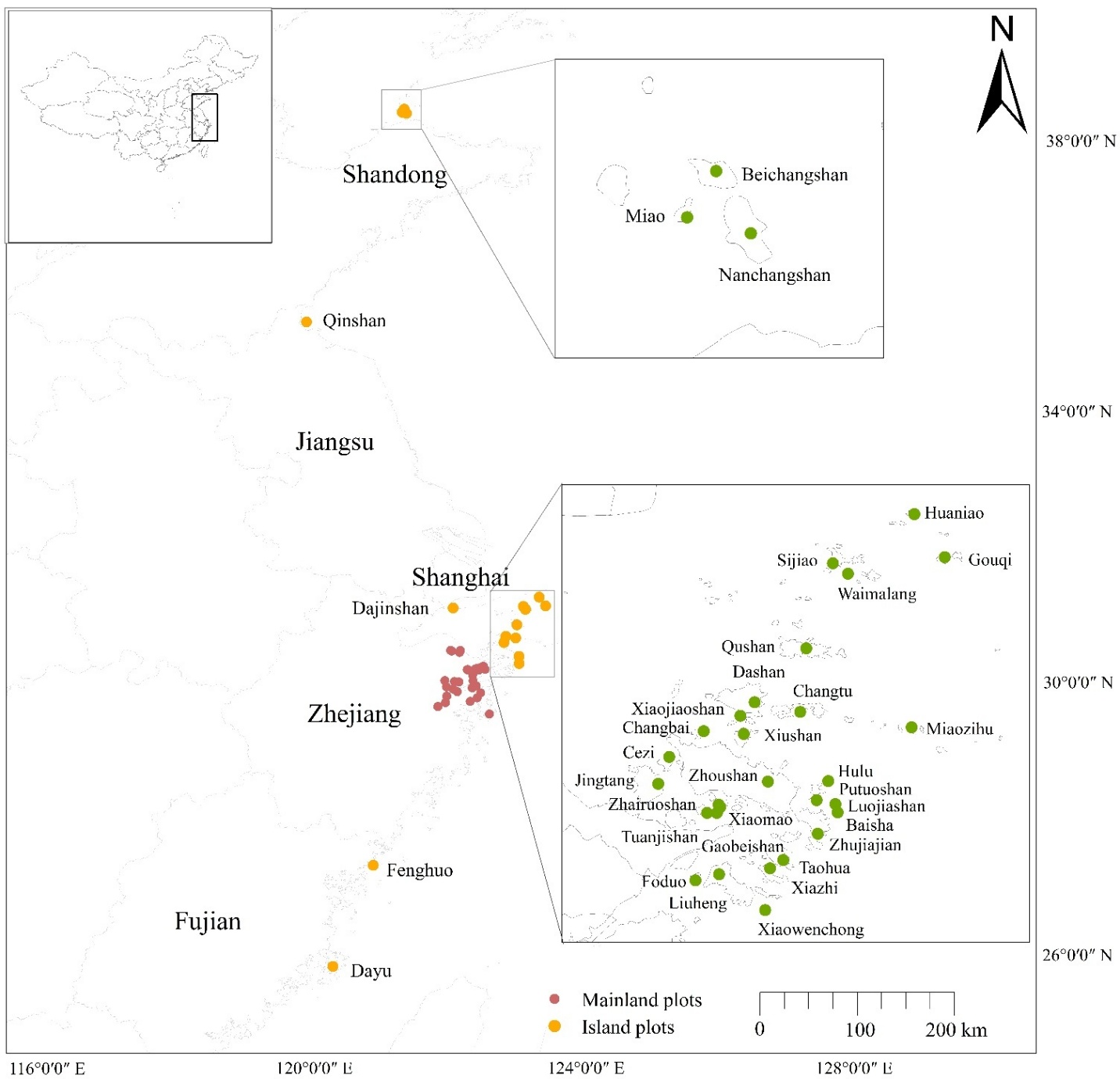


**Fig. S2** Trait collection from each branch. For trait abbreviations, see Table 1. K_s_ and K_l_ are not shown because they are calculated from wood anatomy and leaf traits.


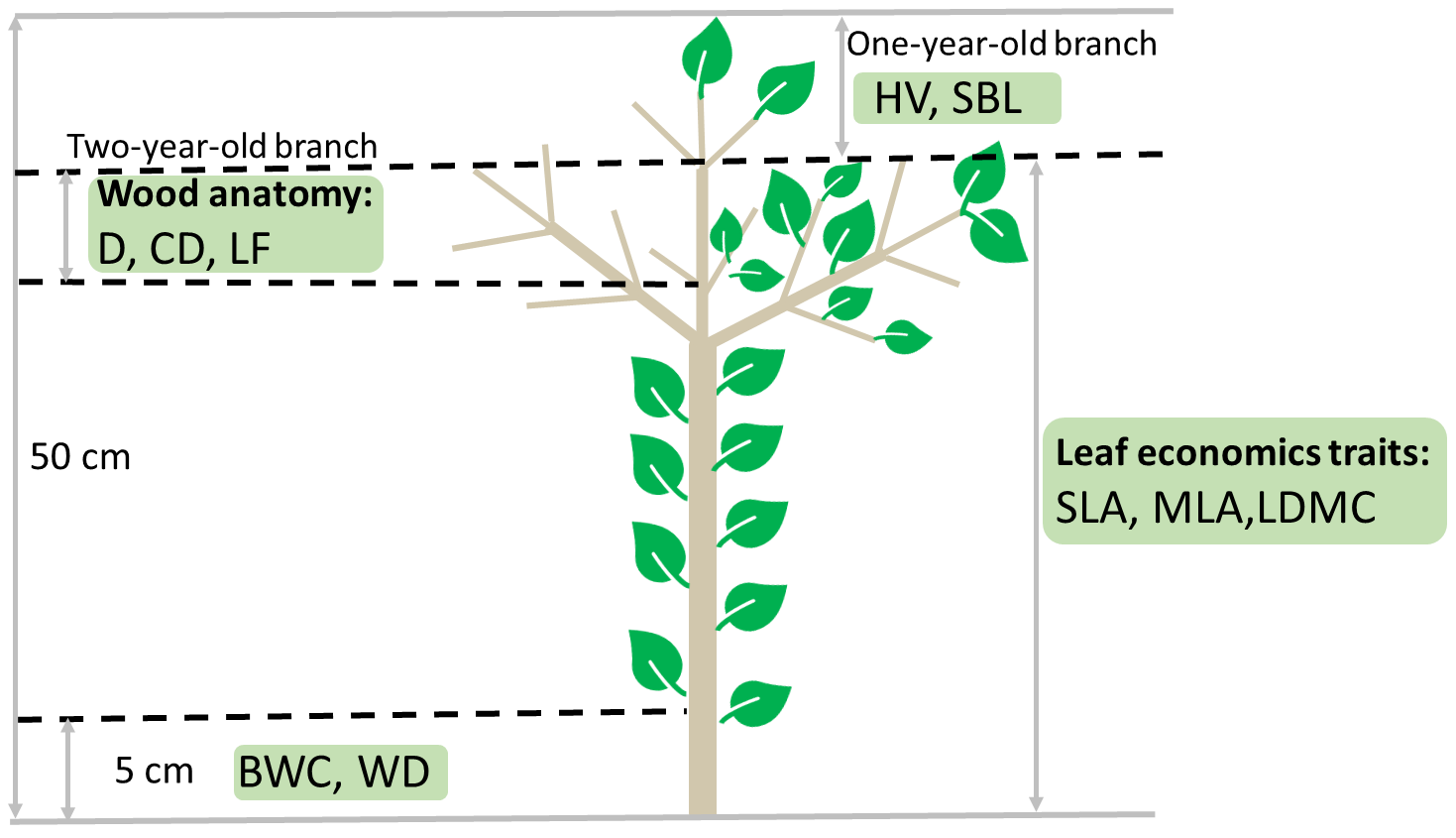


**Fig. S3** Principal components analysis (PCA) for the first two axes (a-c) based on all 13 traits for plants in 16 islands at different organization levels. Individual-level analyses are shown in the left column (n=1143), species-level analyses are shown in the middle column (n=60), and community-level analyses (n=16) are shown in the right column. Black color indicates traits related to leaf and plant size, and leaf toughness, purple indicates wood anatomy and hydraulics and red indicates branch size and density. For trait abbreviations, see Table 1.


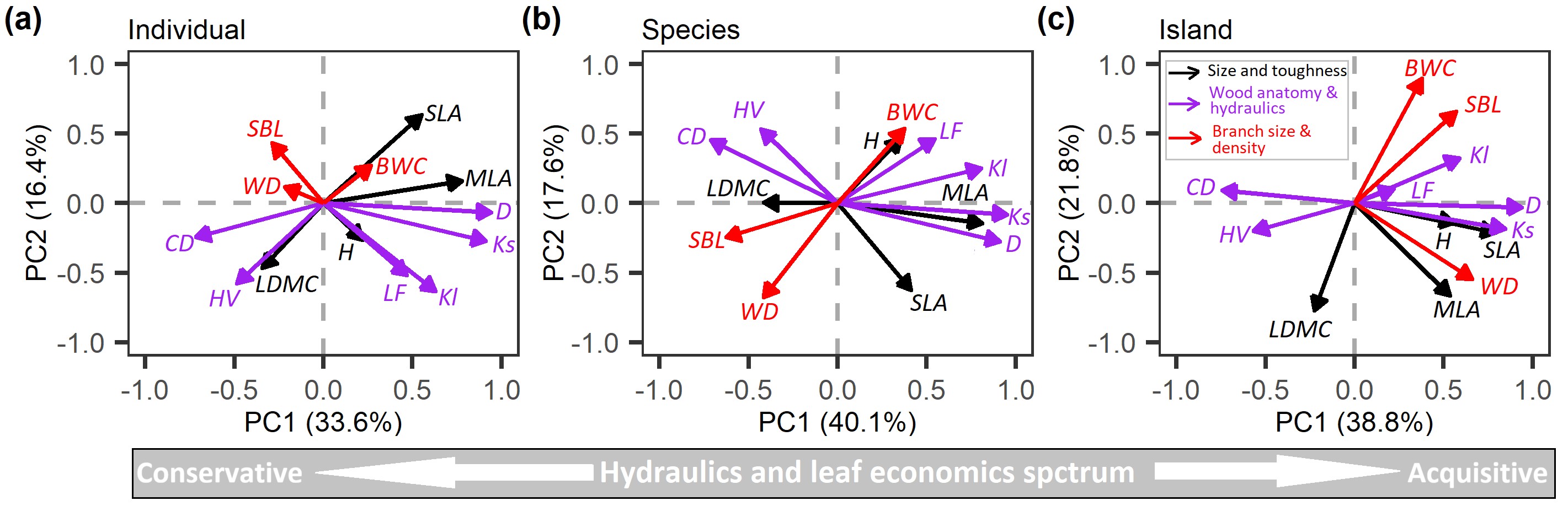


**Fig. S4** Island area and soil properties affect species-level trait associations. The y-axis indicates the related pair-wise trait slope (shown inside each panel). These pair-wise trait slopes correspond to the same trait categories in Fig. 3. WD, wood density; H, tree height; K_s_, xylem hydraulic conductivity; MLA, leaf area; SLA, specific leaf area; LDMC, leaf dry matter content; HV, Huber value; D, conduit diameter; CD, conduit density; LF, lumen fraction. Non-linear regressions reflect log-transformed regressions. R2 and significant regression lines are shown. *, *P*<0.05; **, *P*<0.01.


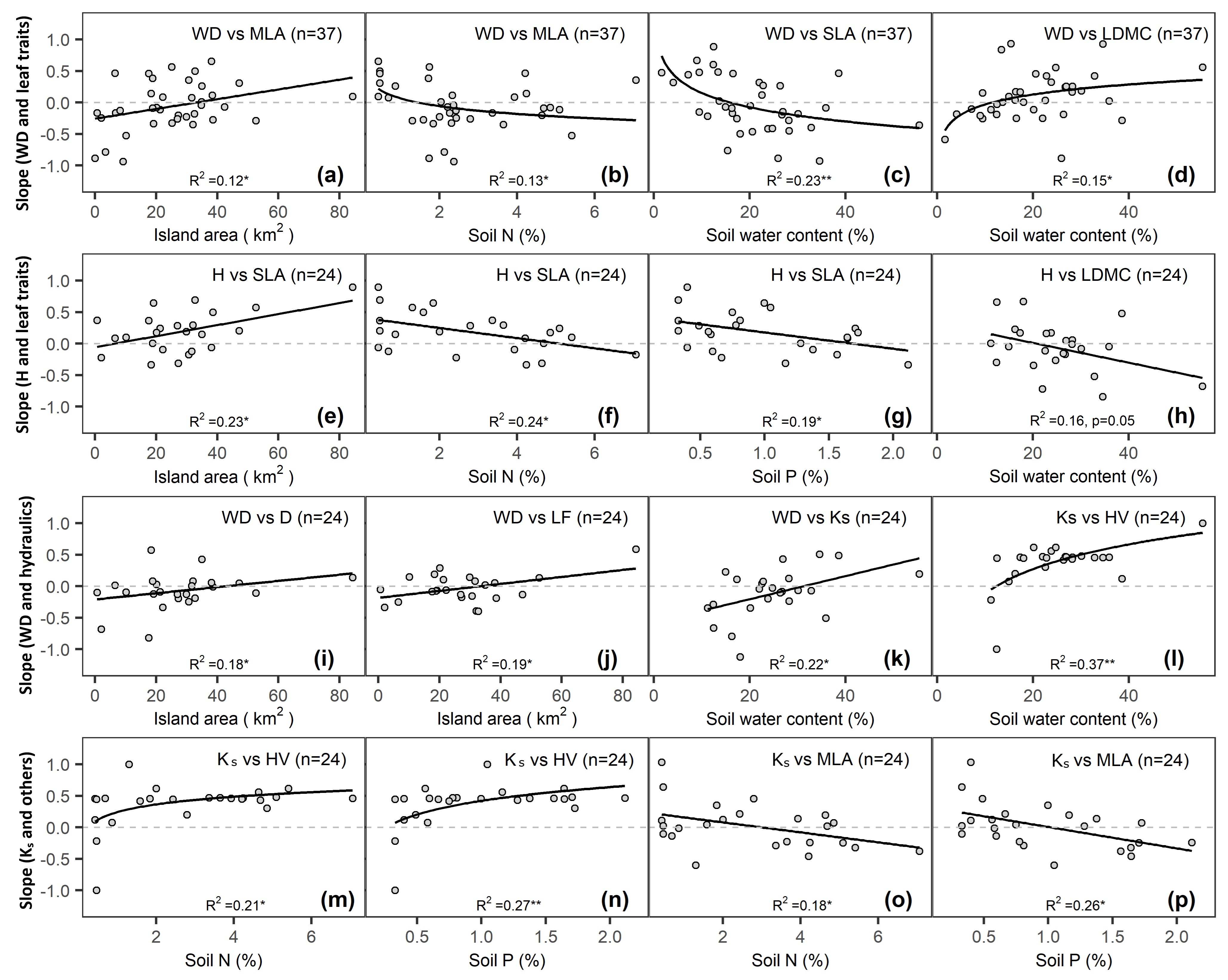


**Fig. S5** The conservative-acquisitive strategies follow the island rule. Insular size changes (y-axis, lnRR=log-transformed (island trait value/mainland trait value)) are shown with conservative-acquisitive strategies (x-axis). The species-specific PC scores were obtained from mainland species in Fig. 4b. **a-c**, tree height (H) and leaf traits. SLA, specific leaf area. LDMC, leaf dry matter content. **d-f**, wood traits for BWC (branch water content), SBL (specific leaf length), and WD (wood density). Fewer than 37 shared species are shown due to missing trait measurements. R^2^ and significant regression lines are shown. *, *P*<0.05; **, *P*<0.01.


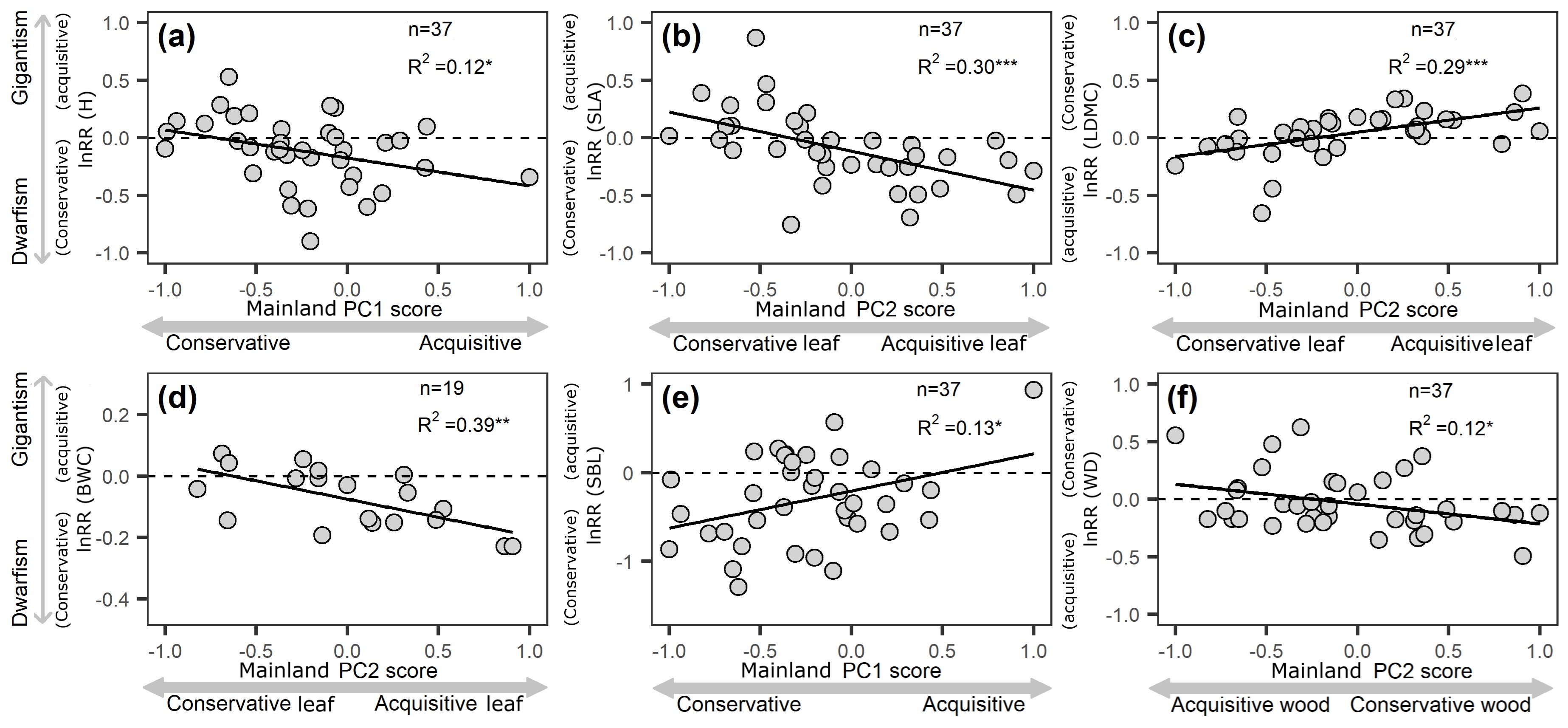


**Fig. S6** The significant impacts of island area, island remoteness, and soil nutrients on island CWM traits (n=10-34). The first row indicates relationships between island biogeographic and CWM traits, and the second row indicates relationships between soil nutrients and CWM traits. **a-c**, island area and CWM traits for tree height (a), conduit density (CD, b), and huber value (HV, c). **d-g**, island remoteness and CWM traits for height (d), specific leaf area (SLA, e), leaf dry matter content (LDMC, f), and conduit diameter (D, g). **h-k**, soil nitrogen (N) and CWM traits for SLA (h), LDMC (i), HV (j), and specific branch length (SBL, k). **i-n**, soil phosphorus (P) and CWM traits for wood anatomy in terms of conduit number and size, respectively. A reduced number of 16 island communities was shown, since some trait and soil data were not collected for all 35 island communities. Regression lines, equations and R^2^ are shown. *, *P*<0.05; **, *P*<0.01.


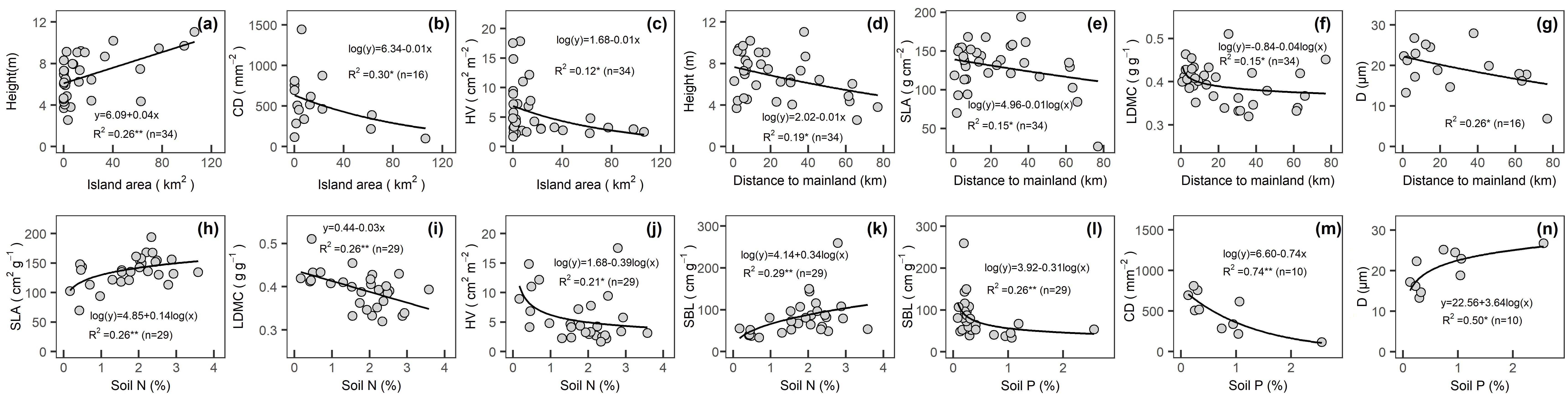


**Fig. S7** Climate data weakly affect species-level trait shifts. The y-axis indicates Insular size changes (y-axis, lnRR=log-transformed (island trait value/mainland trait value)), and the x-axis indicates climate variables. Non-linear regressions reflect log-transformed regressions. R^2^ and significant regression lines are shown. *, *P*<0.05; **, *P*<0.01.


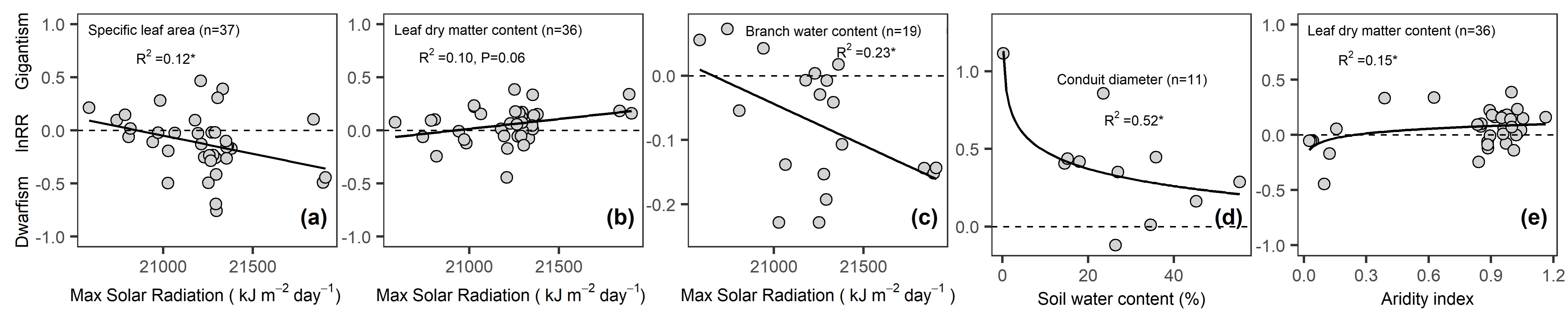


**Table S1** The geographic information of 35 studied islands in eastern China.

| **Names** | **Longitude** | **Latitude** | **Area**  **(km^2^)** | **Remoteness (km)** | **Highest altitude**  **(m)** | **Number of plots** | **Main vegetation types** |
| --- | --- | --- | --- | --- | --- | --- | --- |
| Baisha | 122.45 | 29.94 | 1.60 | 30.50 | 92 | 3 | Evergreen shrub |
| Beichangshan | 120.71 | 37.98 | 7.98 | 14.79 | 195.7 | 4 | Deciduous broad-leaved and coniferous forests, shrubs |
| Cezi | 121.94 | 30.11 | 14.10 | 2.50 | 252.7 | 6 | Deciduous broad-leaved and evergreen broad-leaved forests |
| Changbai | 122.04 | 30.19 | 11.10 | 17.30 | 249.1 | 7 | Deciduous broad-leaved forests |
| Dachangtu | 122.34 | 30.25 | 33.56 | 38.50 | 288.5 | 7 | Coniferous forest |
| Dajinshan | 121.33 | 30.75 | 0.23 | 6.20 | 103.4 | 14 | Deciduous and evergreen broad-leaved forest |
| Dayu | 119.69 | 25.45 | 0.22 | 1.80 | 45.1 | 6 | Deciduous broad-leaved forest, coniferous forest |
| Daishan | 122.20 | 30.28 | 106.29 | 37.80 | 265.5 | 3 | Deciduous broad-leaved forest |
| Fenghuo | 120.26 | 26.93 | 2.32 | 0.65 | 455.2 | 5 | Coniferous and deciduous broad-leaved forests |
| Fodu | 122.02 | 29.74 | 7.30 | 7.80 | 150 | 5 | Deciduous broad-leaved and evergreen broad-leaved forests |
| Gaobeishanyu | 122.09 | 29.97 | 0.0062 | 7.50 | 12.5 | 1 | Evergreen shrub |
| Gouji | 122.78 | 30.72 | 5.85 | 77.00 | 199.3 | 1 | Coniferous forest and shrubland |
| Huaniao | 122.68 | 30.85 | 3.59 | 66.00 | 236.9 | 3 | Deciduous broad-leaved forest, coniferous and evergreen shrub |
| Hulu | 122.42 | 30.04 | 0.931 | 31.50 | 89 | 3 | Evergreen shrub |
| Jintang | 121.91 | 30.03 | 77.30 | 3.20 | 455.7 | 10 | Deciduous broad-leaved and evergreen broad-leaved forests |
| Liuheng | 122.09 | 29.75 | 97.9 | 5.30 | 300.1 | 10 | Deciduous broad-leaved and evergreen broad-leaved forests |
| Luojiashan | 122.44 | 29.97 | 0.38 | 30.6 | 101.7 | 6 | Evergreen broad-leaved forest |
| Miao | 120.68 | 37.94 | 1.425 | 12.34 | 98.3 | 4 | Coniferous and deciduous broad-leaved forests |
| Miaozihu | 122.67 | 30.20 | 2.65 | 61.50 | 142.5 | 3 | Evergreen shrub |
| Nanchangshan | 120.74 | 37.92 | 13.21 | 6.86 | 156.1 | 3 | Coniferous and deciduous broad-leaved forests |
| Putuoshan | 122.39 | 29.98 | 12.5 | 25.30 | 288.2 | 1 | Coniferous forest |
| Qinshan | 119.28 | 34.87 | 0.18 | 6.60 | 45 | 4 | Coniferous and deciduous broad-leaved forests |
| Qushan | 122.36 | 30.44 | 62.85 | 62.07 | 319 | 3 | Coniferous, deciduous broad-leaved and evergreen broad-leaved forests |
| Sijiao | 122.44 | 30.71 | 22.66 | 45.90 | 217.9 | 2 | Evergreen broad-leaved forest |
| Taohua | 122.29 | 29.80 | 40.40 | 9.20 | 544.7 | 8 | Evergreen broad-leaved forests and shrubs |
| Waimalang | 122.48 | 30.67 | 0.18 | 63.50 | 59.7 | 3 | Coniferous and deciduous broad-leaved forests |
| Xiaojiaoshan | 122.16 | 30.24 | 0.16 | 36.00 | 57.3 | 1 | Deciduous broad-leaved forest |
| Xiaomanshan | 122.06 | 29.94 | 0.537 | 5.70 | 126.9 | 3 | Deciduous broad-leaved and evergreen broad-leaved forests |
| Xiaotuanjishan | 122.10 | 29.96 | 0.0061 | 5.80 | 18.2 | 3 | Evergreen shrub |
| Xiaowenchong | 122.23 | 29.65 | 0.27 | 23.10 | 88.6 | 2 | Evergreen shrub |
| Xiazhi | 122.25 | 29.78 | 17.3 | 13.60 | 207.4 | 7 | Evergreen broad-leaved forests |
| Xiushan | 122.17 | 30.18 | 22.66 | 35.81 | 192.4 | 2 | Coniferous and deciduous forests |
| Zhairuoshan | 122.09 | 29.94 | 2.4 | 4.50 | 215.4 | 3 | Coniferous and evergreen broad-leaved forests |
| Zhoushan | 122.24 | 30.04 | 490.90 | 8.80 | 503.5 | 4 | Evergreen broad-leaved forests |
| Zhujiajian | 122.39 | 29.88 | 62.20 | 18.60 | 376.6 | 2 | Evergreen broad-leaved forest |

Note: Remoteness was quantified as the distance from the island to the nearest mainland (Xu *et al.*, 2023). Remoteness and island area data were obtained from China Island Records (2013). When remoteness was not available, it was obtained from Xu *et al.* (2023).

**Table S2** 37 shared species studied in both the island and mainland sites.

| Family | Genera | species |
| --- | --- | --- |
| Anacardiaceae | *Rhus* | *Rhus chinensis* Mill. |
| Aquifoliaceae | *Ilex* | *Ilex chinensis* Sims |
| Aquifoliaceae | *Ilex* | *Ilex rotunda* Thunb. |
| Cannabaceae | *Celtis* | *Celtis sinensis* Pers. |
| Ericaceae | *Rhododendron* | *Rhododendron simsii* Planch. |
| Ericaceae | *Vaccinium* | *Vaccinium bracteatum* Thunb. |
| Euphorbiaceae | *Mallotus* | *Mallotus japonicus* (L.f.) Müll.Arg. |
| Euphorbiaceae | *Vernicia* | *Vernicia fordii* (Hemsl.) Airy Shaw |
| Fagaceae | *Cyclobalanopsis* | *Cyclobalanopsis glauca* Oerst. |
| Fagaceae | *Lithocarpus* | *Lithocarpus glaber* Nakai |
| Fagaceae | *Quercus* | *Quercus acutissima* Carruth. |
| Fagaceae | *Quercus* | *Quercus fabri* Hance |
| Hamamelidaceae | *Loropetalum* | *Loropetalum chinense* (R.Br.) Oliv. |
| Juglandaceae | *Platycarya* | *Platycarya strobilacea* Siebold & Zucc. |
| Leguminosae | *Albizia* | *Albizia kalkora* (Roxb.) Prain |
| Leguminosae | *Dalbergia* | *Dalbergia hupeana* Hance |
| Lauraceae | *Camphora* | *Camphora officinarum* Nees |
| Lauraceae | *Cinnamomum* | *Cinnamomum japonicum* Siebold |
| Lauraceae | *Lindera* | *Lindera glauca* (Siebold & Zucc.) Blume |
| Lauraceae | *Litsea* | *Litsea coreana* var. *sinensis* (C.K.Allen) Yen C.Yang & P.H.Huang |
| Lauraceae | *Machilus* | *Machilus thunbergii* Siebold & Zucc. |
| Lamiaceae | *Clerodendrum* | *Clerodendrum cyrtophyllum* Turcz. |
| Moraceae | *Ficus* | *Ficus erecta* Thunb. |
| Myricaceae | *Morella* | *Morella rubra* Lour. |
| Myrtaceae | *Syzygium* | *Syzygium buxifolium* Hook. & Arn. |
| Pentaphylacaceae | *Eurya* | *Eurya japonica* Thunb. |
| Pinaceae | *Pinus* | *Pinus massoniana* Siebold & Zucc. |
| Rosaceae | *Rhaphiolepis* | *Rhaphiolepis indica* (L.) Lindl. |
| Rubiaceae | *Gardenia* | *Gardenia jasminoides* J.Ellis |
| Rutaceae | *Zanthoxylum* | *Zanthoxylum ailanthoides* Siebold & Zucc. |
| Theaceae | *Schima* | *Schima superba* Gardner & Champ. |
| Staphyleaceae | *Euscaphis* | *Euscaphis japonica* (Thunb.) Kanitz |
| Styracaceae | *Styrax* | *Styrax confusus* Hemsl. |
| Symplocaceae | *Symplocos* | *Symplocos setchuensis* Brand ex Diels |
| Symplocaceae | *Symplocos* | *Symplocos stellaris* Brand |
| Symplocaceae | *Symplocos* | *Symplocos sumuntia* Buch.-Ham. ex D.Don |
| Symplocaceae | *Symplocos* | *Symplocos tanakana* Nakai |

**Table S3** Bivariate Pearson correlations among 7 studied traits in islands and mainland at species (n=37 in grey) and community level (n=35 or 66 in green). Bold coefficients indicate P<0.05. Underlined coefficients indicate adjusted P<0.05.

|  | **H** | **MLA** | **SLA** | **LDMC** | **HV** | **WD** |
| --- | --- | --- | --- | --- | --- | --- |
| **Island species (n=37)** | | | | | | |
| **MLA** | 0.11 |  |  |  |  |  |
| **SLA** | -0.21 | 0.28 |  |  |  |  |
| **LDMC** | **0.35** | -0.22 | **-0.64** |  |  |  |
| **HV** | -0.07 | **-0.42** | -0.23 | -0.10 |  |  |
| **WD** | 0.01 | -0.30 | **-0.33** | **0.51** | -0.13 |  |
| **SBL** | -0.20 | **-0.62** | 0.20 | 0.03 | 0.01 | 0.30 |
| **Island communities (n=35)** | | | | | | |
| **MLA** | **0.45** |  |  |  |  |  |
| **SLA** | **0.60** | **0.56** |  |  |  |  |
| **LDMC** | 0.08 | 0.10 | **-0.42** |  |  |  |
| **HV** | **-0.45** | -0.31 | **-0.65** | **0.35** |  |  |
| **WD** | 0.24 | **0.65** | **0.67** | **-0.35** | **-0.44** |  |
| **SBL** | 0.29 | -0.22 | **0.40** | -0.26 | -0.25 | 0.06 |
| **Mainland species (n=37)** | | | | | | |
| **MLA** | **0.34** |  |  |  |  |  |
| **SLA** | -0.17 | 0.11 |  |  |  |  |
| **LDMC** | 0.06 | **-0.33** | **-0.60** |  |  |  |
| **HV** | 0.02 | -0.07 | -0.28 | 0.04 |  |  |
| **WD** | -0.04 | -0.10 | 0.07 | -0.12 | -0.13 |  |
| **SBL** | -0.29 | **-0.73** | 0.19 | 0.23 | -0.09 | 0.29 |
| **Mainland communities (n=66)** | | | | | | |
| **MLA** | **0.43** |  |  |  |  |  |
| **SLA** | 0.19 | **0.43** |  |  |  |  |
| **LDMC** | **-0.28** | **-0.42** | **-0.32** |  |  |  |
| **HV** | **-0.25** | **-0.45** | **-0.25** | 0.21 |  |  |
| **WD** | 0.09 | 0.24 | 0.22 | -0.19 | **-0.40** |  |
| **SBL** | 0.08 | -0.19 | -0.06 | -0.10 | -0.11 | 0.09 |

Note: MLA, leaf area; SLA, specific leaf area; LDMC, leaf dry matter content;HV, Huber value; WD, wood density; SBL, specific branch length.

**Table S4** Bivariate Pearson correlations among 13 complete traits in 16 islands at individual (n=1143), species (n=60), and community level (n=16). Bold coefficients indicate P<0.05. Underlined coefficients indicate adjusted P<0.05.

|  | **H** | **MLA** | **SLA** | **LDMC** | **K_s_** | **K_l_** | **HV** | **D** | **CD** | **LF** | **WD** | **BWC** |
| --- | --- | --- | --- | --- | --- | --- | --- | --- | --- | --- | --- | --- |
| **Individual** | | | | | | | | | | | | |
| **MLA** | **0.12** |  |  |  |  |  |  |  |  |  |  |  |
| **SLA** | **-0.07** | **0.47** |  |  |  |  |  |  |  |  |  |  |
| **LDMC** | **0.06** | **-0.22** | **-0.55** |  |  |  |  |  |  |  |  |  |
| **K_s_** | **0.18** | **0.58** | **0.33** | **-0.15** |  |  |  |  |  |  |  |  |
| **K_l_** | **0.16** | **0.24** | 0.02 | -0.05 | **0.78** |  |  |  |  |  |  |  |
| **HV** | **-0.08** | **-0.53** | **-0.54** | **0.18** | **-0.33** | **0.26** |  |  |  |  |  |  |
| **D** | **0.17** | **0.64** | **0.42** | **-0.18** | **0.92** | **0.68** | **-0.40** |  |  |  |  |  |
| **CD** | **-0.08** | **-0.56** | **-0.42** | **0.17** | **-0.55** | **-0.34** | **0.37** | **-0.83** |  |  |  |  |
| **LF** | **0.18** | **0.21** | 0.05 | -0.04 | **0.62** | **0.60** | **-0.11** | **0.35** | **0.16** |  |  |  |
| **WD** | **-0.08** | **-0.16** | -0.02 | **0.15** | **-0.15** | **-0.13** | 0.00 | **-0.10** | -0.01 | **-0.18** |  |  |
| **BWC** | **0.10** | **0.19** | **0.19** | **-0.37** | **0.14** | 0.06 | **-0.14** | **0.14** | **-0.11** | **0.08** | **-0.28** |  |
| **SBL** | **-0.16** | **-0.44** | **0.15** | -0.02 | **-0.24** | **-0.25** | **-0.10** | **-0.20** | **0.11** | **-0.19** | **0.17** | **0.13** |
| **Species** | | | | | | | | | | | | |
| **MLA** | 0.12 |  |  |  |  |  |  |  |  |  |  |  |
| **SLA** | -0.19 | **0.43** |  |  |  |  |  |  |  |  |  |  |
| **LDMC** | 0.01 | -0.24 | **-0.45** |  |  |  |  |  |  |  |  |  |
| **K_s_** | **0.33** | **0.71** | **0.36** | **-0.30** |  |  |  |  |  |  |  |  |
| **K_l_** | **0.34** | **0.49** | 0.08 | **-0.29** | **0.84** |  |  |  |  |  |  |  |
| **HV** | -0.06 | **-0.46** | **-0.55** | 0.08 | **-0.45** | 0.08 |  |  |  |  |  |  |
| **D** | **0.26** | **0.69** | **0.42** | **-0.28** | **0.95** | **0.76** | **-0.46** |  |  |  |  |  |
| **CD** | -0.10 | **-0.55** | **-0.38** | 0.24 | **-0.74** | **-0.59** | **0.36** | **-0.91** |  |  |  |  |
| **LF** | **0.34** | **0.37** | 0.06 | -0.18 | **0.56** | **0.51** | -0.18 | **0.29** | 0.08 |  |  |  |
| **WD** | **-0.28** | **-0.29** | 0.14 | 0.21 | **-0.30** | **-0.36** | -0.06 | -0.17 | 0.05 | **-0.45** |  |  |
| **BWC** | **0.36** | 0.18 | 0.02 | **-0.43** | 0.25 | **0.31** | 0.01 | 0.18 | -0.09 | **0.28** | **-0.63** |  |
| **SBL** | -0.20 | **-0.72** | 0.00 | 0.12 | **-0.52** | **-0.53** | 0.04 | **-0.45** | **0.29** | **-0.46** | **0.38** | -0.04 |
| **Community** | | | | | | | | | | |  |  |
| **MLA** | **0.65** |  |  |  |  |  |  |  |  |  |  |  |
| **SLA** | **0.64** | **0.75** |  |  |  |  |  |  |  |  |  |  |
| **LDMC** | 0.11 | 0.21 |  |  |  |  |  |  |  |  |  |  |
| **K_s_** | **0.59** | **0.65** | **0.69** | -0.09 |  |  |  |  |  |  |  |  |
| **K_l_** | 0.05 | 0.07 | 0.08 | -0.32 | **0.53** |  |  |  |  |  |  |  |
| **HV** | -0.32 | -0.26 | **-0.64** | 0.24 | **-0.59** | -0.02 |  |  |  |  |  |  |
| **D** | **0.61** | **0.58** | **0.74** | -0.18 | **0.89** | 0.46 | **-0.65** |  |  |  |  |  |
| **CD** | -0.44 | -0.49 | **-0.76** | 0.17 | **-0.57** | -0.21 | **0.72** | **-0.81** |  |  |  |  |
| **LF** | 0.12 | 0.22 | 0.12 | -0.20 | 0.50 | 0.55 | 0.00 | 0.28 | 0.11 |  |  |  |
| **WD** | 0.41 | **0.84** | **0.73** | 0.03 | **0.69** | 0.31 | -0.47 | **0.72** | **-0.73** | 0.19 |  |  |
| **BWC** | 0.08 | -0.32 | 0.09 | **-0.67** | 0.20 | 0.41 | -0.48 | 0.25 | -0.10 | 0.41 | -0.19 |  |
| **SBL** | 0.24 | -0.02 | 0.43 | -0.39 | 0.37 | 0.36 | **-0.63** | 0.38 | -0.26 | 0.40 | 0.08 | **0.82** |

Note: MLA, leaf area; SLA, specific leaf area; LDMC, leaf dry matter content; K_s_, xylem hydraulic conductivity; K_l_, leaf hydraulic conductivity; HV, Huber value; D, conduit diameter; CD, conduit density; LF, lumen fraction; WD, wood density; BWC, branch water content; SBL, specific branch length.

**Table S5** Fitted equations and corresponding formulas for each significant regression line in Fig. 6.

| **Formulas** | **Equation** |
| --- | --- |
| (a): Wood density (WD)~mean leaf area (MLA) | Island species: log(y)=-0.58-0.001x  Island community: log(y)=-0.81+log(x)  Mainland community: log(y)=-11.36+log(x) |
| (b): Wood density (WD)~specific leaf area (SLA) | Island species: y=0.59-0.0003x  Island community: y=0.45+0.0008x |
| (c): Wood density (WD)~leaf dry matter content (LDMC) | Island species: y=0.37+0.44x  Island community: y=-0.69-0.32x |
| (d): Wood density (WD)~Huber value (HV) | Island community: y=-0.511-0.05log(x)  Mainland community: y=0.73-0.20log(x) |
| (e):Height (H)~mean leaf area (MLA) | Island community: log(y)=1.05+0.25log(x)  Mainland species: log(y)=1.29+0.004x  Mainland community: y=6.70+0.12x |
| (f): Height (H)~specific leaf area (SLA) | Island community: log(y)=0.95+0.007x |
| (g): Height (H)~leaf dry matter content (LDMC) | Mainland community: y=19.11-21.84x |
| (h): Height (H)~Huber value (HV) | Island community: log(y)=2.25-0.25log(x)  Mainland community: y=12.12-0.63x |
| (i): Wood density (WD)~hydraulic conductivity (K_s_) | Island species: y=0.54-0.03log(x)  Island community: log(y)=-0.59+0.08log(x) |
| (j): Wood density (WD)~conduit diameter (D) | Island community: log(y)=-1.08+0.16log(x) |
| (k): Wood density (WD)~conduit density (CD) | Island community: y=0.59-0.000086x |
| (l): Wood density (WD)~lumen fraction (LF) | Island species: y=0.29-0.09log(x) |
| (m) Hydraulic conductivity (K_s_)~height (H) | Island species: log(y)=-1.54+0.96log(x)  Island community: y=0.17+0.13x |
| (n) Hydraulic conductivity (K_s_)~mean leaf area (MLA) | Island species: y=-1.24+0.67log(x)  Island community: log(y)=-1.95+0.54log(x) |
| (o) Hydraulic conductivity (K_s_)~specific leaf area (SLA) | Island species: y=-6.09+1.47log(x)  Island community: log(y)=-77.28+11.51log(x)  Mainland species: log(y)=1.60-0.009x |
| (p) Hydraulic conductivity (K_s_)~Huber value (HV) | Island community: log(y)=0.43-0.09x |

**Table S6** Results of median-centered Levene’s tests comparing trait variance between island and mainland species. *P-values* indicate significant differences (*P*<0.05). Traits with insufficient variation in one group (e.g. K_s_, CD) could not be tested with Levene’s test.

|  | **Trait** | **df** | **F-value** | ***P-value*** | **Interpretation** |
| --- | --- | --- | --- | --- | --- |
| Species level | H | 1, 36 | 1.93 | 0.17 | No difference |
|  | MLA | 1, 36 | 0.04 | 0.84 | No difference |
|  | SLA | 1, 36 | <0.001 | 0.99 | No difference |
|  | LDMC | 1, 36 | 0.83 | 0.37 | No difference |
|  | HV | 1, 36 | 0.17 | 0.69 | No difference |
|  | **WD** | 1, 36 | 6.03 | **0.02** | **Lower variance on islands** |
|  | SBL | 1, 36 | 1.11 | 0.30 | No difference |
|  | **K_s_** | 1, 15 | 4.65 | **0.048** | **Lower variance on islands** |
|  | Kl | 1, 15 | 2.52 | 0.13 | No difference |
|  | D | 1, 15 | 1.11 | 0.31 | No difference |
|  | **CD** | 1, 15 | 9.60 | **0.007** | **Lower variance on islands** |
|  | BWC | 1, 20 | 0.38 | 0.54 | No difference |

Note: H, tree height; MLA, leaf area; SLA, specific leaf area; LDMC, leaf dry matter content; HV, Huber value; WD, wood density; SBL, specific branch length; K_s_, xylem hydraulic conductivity; K_l_, leaf hydraulic conductivity; D, conduit diameter; CD, conduit density; BWC, branch water content.

**Methods S1** Environment data collection

For each plot, we recorded the longitude and latitude in the field. To quantify climate variables related to water deficit, heat and cold, light and wind conditions, we extracted climate data from WordClim 2.0 (<https://www.worldclim.org/>) with a resolution of 1 km^2^. Water deficit refers to mean annual precipitation (MAP, mm), mean annual evapotranspiration (PET), mean vapor pressure deficit (VPD, kpa), and mean annual aridity index (MAI, MAI=MAP/PET). Heat and cold indices include mean annual temperature (MAT, ℃), minimum (T_min_, ℃) and maximum temperature (T_max_, ℃). Light index refers to maximum solar radiation (S_max_, kJ m^-2^ d^-1^). We quantified wind conditions using mean annual wind speed (V, m s^-1^).

**Methods S2** Analyses of soil and biogeographic influences on trait shifts

To quantify soil characters, we used a metallic corer (20 cm height and 7.5 cm in diameter) and randomly sampled soil from 5 locations in each island plot at a depth of 0-20 cm after removing the litter layer. To exclude the climate impact on soils, we sampled soil on sunny days without rainy records in the past three days. To quantify soil nutrients, we measured soil carbon content (C, %), nitrogen content (N, %), and phosphorus content (P, %). C indicates the degree of soil organic matter and water holding capacity, N indicates soil fertility, and P is related to plant stomata regulation and mechanical resistance (Guillemot *et al.*, 2022). To characterize soil water availability and compaction, we calculated soil water content (SWC, g g^-1^) as soil dry mass to fresh mass, and soil bulk density (BD, g cm^-3^) as soil dry mass over soil volume (Poorter *et al.*, 2021). We used a standardized method to measure the above-mentioned soil variables (He *et al.*, 2021). For each shared species, we calculated mean soil conditions across all sampled islands, forming a species-specific soil niche (Liu *et al.*, 2021). Island area and remoteness were averaged per species and used to test their effects on trait shifts (lnRR), but no significant relationships were detected. Similarly, CWM-level soil and biogeographic niche per community were averaged by islands.

**References**

**2013.** *China Island Records[中国海岛志]*: Ocean Publishing Company.

**Guillemot J, Martin‐StPaul NK, Bulascoschi L, Poorter L, Morin X, Pinho BX, Le Maire G, RL Bittencourt P, Oliveira RS, Bongers FJGCB. 2022.** Small and slow is safe: On the drought tolerance of tropical tree species. **28**(8): 2622-2638.

**He D, Biswas SR, Xu MS, Yang TH, You WH, Yan ER. 2021.** The importance of intraspecific trait variability in promoting functional niche dimensionality. *Ecography* **44**(3): 380-390.

**Liu Q, Sterck FJ, Zhang J-L, Scheire A, Konings E, Cao M, Sha L-Q, Poorter L. 2021.** Traits, strategies, and niches of liana species in a tropical seasonal rainforest. *Oecologia* **196**: 499-514.

**Poorter L, Craven D, Jakovac CC, van der Sande MT, Amissah L, Bongers F, Chazdon RL, Farrior CE, Kambach S, Meave JA. 2021.** Multidimensional tropical forest recovery. *Science* **374**(6573): 1370-1376.

**Xu M, Yang A, Yang X, Cao W, Zhang Z, Li Z, Zhang Y, Zhang H, You W, Yan ER. 2023.** Island area and remoteness shape plant and soil bacterial diversity through land use and biological invasion. *Functional Ecology* **37**(5): 1232-1244.
